# The economic impacts of COVID-19 hospitalizations, intensive care unit admissions, and deaths related to overweight and obesity

**DOI:** 10.1371/journal.pgph.0001445

**Published:** 2025-06-04

**Authors:** Adeyemi Okunogbe, Donal Bisanzio, Garrison Spencer, Shradha Chhabria, Jaynaide Powis, Rachel Nugent

**Affiliations:** 1 RAND Corporation, Arlington, Virginia, United States of America; 2 RTI International, Durham, North Carolina, United States of America; 3 Departments of Internal Medicine and Pediatrics, University Hospitals Cleveland Medical Center and Rainbow Babies and Children’s Hospital, Cleveland, Ohio, United States of America; 4 World Obesity Federation, London, United Kingdom; PLOS: Public Library of Science, UNITED KINGDOM OF GREAT BRITAIN AND NORTHERN IRELAND

## Abstract

During the COVID-19 pandemic, it quickly became clear that people living with overweight and obesity (OAO) have a higher risk for more severe health outcomes. The objective of this study is to investigate how the health and economic impacts of COVID-19 are exacerbated by OAO. We estimated economic impacts of COVID-19 associated with OAO for eight countries using a cost-of-illness approach from a limited societal perspective. Direct medical costs and premature mortality costs between 2020 and 2030 were estimated. Country-specific data were sourced from published studies and global databases. Additional COVID-19 hospitalizations, ICU admissions, and deaths among the population with OAO accounted for approximately 20% of hospitalizations, 43% of ICU admissions, and 17% of deaths from COVID-19 in 2020 and 2021 on average across the eight countries. As a percent of GDP, additional treatment and premature mortality costs ranged from between 0.0003% in Thailand to 0.62% in Brazil in 2020 and between 0.009% in Australia to 0.56% in Brazil in 2021. In future COVID-19 prevalence scenarios, keeping OAO prevalence at 2019 levels or reducing it by 50% will translate into average annual reductions of 17.4%-18.5% and 40.8%-41.4% in additional costs respectively between 2022 and 2030 across the eight countries. This study provides initial evidence on the significant economic impacts of COVID-19 on populations with OAO. Our findings support the need for strengthened political commitment and adequate prioritization of OAO prevention and reduction interventions to help increase resilience to public health emergencies in these and other countries.

## Introduction

In addition to the immense loss of life caused by COVID-19 – 7.0 million deaths as of February 2025 – the pandemic has exerted a heavy economic toll [1]. According to the International Monetary Fund (IMF), in part due to the US$16.5 trillion in fiscal measures that have been committed to fight the pandemic, global government debt has reached an unprecedented level of nearly 100% of global GDP [2]. Global economic output fell by 3.2% in 2020 and nearly 80 million additional people were likely to enter extreme poverty in 2020–2021 compared to pre-pandemic projections [2].

There are numerous pathways through which COVID-19 affects health and economic outcomes. One of these critical pathways is through its impact on non-communicable diseases (NCDs). In New Zealand, Nghiem and Wilson estimated that the increased risk of cardiovascular disease (CVD) associated with unemployment resulting from the pandemic led to an additional US$ 209–346 million in healthcare costs [3]. In the United Kingdom, delays in cancer diagnosis resulting from the pandemic were estimated to cause 32,700 lost quality-adjusted life years (QALYs) and GBP 103.8 million in productivity losses [4]. Using primary data on the relative risk of hospitalization, intensive care unit (ICU) admission, and ventilator support for patients with diabetes, Bain et al. estimated that in Europe, the average cost per hospital admission from COVID-19 ranged from EUR 16,993 for patients without diabetes, to EUR 25,018 for those with type 2 diabetes and good glycemic control, and up to EUR 57,244 for those with type 1 diabetes and poor glycemic control [5]. Another study estimated that treatment of people with overweight and obesity (OAO) that were hospitalized for COVID-19 accounted for 76% of the total direct costs of secondary care during the first wave of COVID-19 in Europe [5]. With OAO being an important risk factor for NCDs, this underscores the additional economic burden placed on patients with an OAO-related condition during the COVID-19 pandemic.

In 2019, the year before the COVID-19 pandemic, there were over 5 million deaths attributable to OAO across the globe from numerous related NCDs including stroke, asthma, diabetes, Alzheimer’s disease, and cancer [6]. As the world began to grapple with the spread of the SARS-CoV-2 virus, it quickly became clear that those living with OAO were at higher risk for hospitalization, poorer outcomes, and death [7–10]. OAO is a condition that does not receive adequate prioritization relative to its growing prevalence and impact, as a risk factor for NCDs and, as we are now acutely aware, infectious disease.

OAO has been shown to have significant economic impacts globally. A recent study of 161 countries estimated an economic cost of about 2.2% of global GDP in 2019 [11]. A study of 33 OECD countries estimated that GDP will be lower by an average of 3.3% between 2020 and 2050 due to OAO [12]. However, while the economic impacts of COVID-19 and OAO have been established independently, not much is known about the economic impacts of COVID-19 associated with OAO.

This study aims to address this gap by quantifying how OAO has worsened the health and economic impacts of COVID-19 and demonstrate how addressing OAO could lead to a healthier and more resilient future. It builds on recent work that estimated the economic impacts of OAO for eight countries - Australia, Brazil, India, Mexico, Saudi Arabia, South Africa, Spain and Thailand - representing a range of geographies and income levels [13,14]. The economic loss due to OAO among those eight countries was estimated to be between 0.8% and 2.4% of GDP in 2019.

This study therefore adds to the evidence base by estimating the COVID-19 costs related to OAO. Quantifying the magnitude of these economic impacts helps policy makers and other stakeholders better understand the scope of the challenge, supports prioritization efforts, and provides a crucial tool for advocacy to urge policy makers to respond effectively. In doing so, the study promotes breaking down the artificial distinction between communicable and chronic diseases which fails to recognize the common risk factors and frequent comorbidities experienced by populations from these two disease categories. Because those living with OAO are at a higher risk of various types of infections and of developing complications, the study also emphasizes the importance of addressing OAO as a health system preparedness and resiliency issue considering that COVID-19 is only the most recent respiratory viral disease to sweep the globe and future infectious disease outbreaks may follow similar patterns.

The COVID-19 pandemic has both exposed the vulnerabilities in our public health systems and demonstrated our ability to rise to a challenge and take collective action. As nations continue to recover and adapt, there is an opportunity to give OAO the policy attention and resource prioritization it requires. Doing so will help ensure a healthier future and increase resilience in the face of future pandemics.

## Methodology

### Study design

We employed a cost-of-illness approach to estimate the OAO-related costs of COVID-19 in 2020 across eight countries. The eight countries included in the analysis (Australia, Brazil, India, Mexico, Saudi Arabia, South Africa, Spain and Thailand) correspond to a previous analysis of economic impacts of OAO published by the authors and were originally selected to represent a range of geographies and income levels and for which adequate data was available [14]. We also projected costs from 2021 to 2030 using a variety of COVID-19 prevalence scenarios to account for varying mitigating strategies such as mask mandates and vaccine roll-out and uptake. Adopting a limited societal perspective, we calculated the OAO-related cost of COVID-19 as the sum of the medical cost of treatment (direct costs) and premature mortality costs (indirect costs). Other costs such as non-medical direct costs (e.g., transportation and informal caregiver costs) and indirect costs such as productivity losses from absenteeism and presenteeism are not included, due to the lack of evidence on the differential impact of COVID-19 on these parameters for population with OAO.

### Data sources

Data for model parameters were sourced from peer-reviewed literature and publicly available global databases (Table A of S1 Table). Country-specific cost of hospitalization and ICU care for COVID-19 patients were drawn from a review of literature. Where country-specific literature was not available for outpatient, hospitalization, and/or ICU costs, the Institute for Health Metrics and Evaluation’s (IHME) estimates of inpatient and outpatient unit costs were used [15]. This was the case for Saudi Arabia and South Africa for which we did not have data for outpatient costs, and India and Thailand for outpatient, hospitalization, and ICU costs. The mean estimate of the cost of an outpatient visit was used for the cost of outpatient visits and the mean estimate of the cost of inpatient visits was used for hospitalization cost. For the cost of ICU admissions, we used the upper bound (95% uncertainty interval) estimate of inpatient visits because the cost of ICU admission was not included in the IHME estimates of healthcare costs. All costs are in 2020 constant US dollars (USD). Cost data was collected in local currency units (LCU) where possible, adjusted for inflation to 2020 values, and converted to USD using average annual exchange rates.

Data on historical OAO prevalence and GDP per capita were drawn from the NCD Risk Factor Collaboration (NCD-RisC) study [16] and IMF World Economic Outlook Database, respectively. Parameters such as population, life expectancy and background death rates were drawn from the United Nations Population Division (UNPD) [17]. Estimates of COVID-19 hospitalizations, ICU admissions and COVID-19 deaths were drawn from the IHME Database [18].

### Epidemiological modelling

#### Estimating the number of COVID-19 hospitalizations and deaths related to OAO.

The population of study countries was divided into four groups based on body mass index (BMI): population with healthy weight (BMI <25); population with overweight (BMI 25 – <30); population with obesity (BMI 30- <35), and population with severe obesity (BMI ≥ 35). The effect of OAO on the outcomes of SARS-CoV2 infection was extracted from the published scientific literature [19–21]. The number of hospitalizations, deaths, and vaccination coverage as of 15 August 2022, were extracted from the IHME COVID-19 and OurWorldInData dashboards [22]. The number of non-ICU hospitalizations, ICU hospitalizations, and deaths were disaggregated for each BMI group using the results of published studies [19–21]. A scoping review was performed to gather information about the effect of BMI on the outcomes of SARS-CoV-2 infections. The results of the scoping review are reported in Table B of S1 Table.

Using data for 2020 and 2021, we estimated the ratio of non-ICU and ICU hospitalizations, and deaths attributable to each of the four BMI groups. The 2022–2030 estimates of hospitalizations, ICU admissions, and deaths in each BMI group were computed using the projections of OAO prevalence. The number of hospitalizations (non-ICU and ICU) was obtained using the ratio of hospitalized (split between ICU and non-ICU) to all cases from IHME data. For projections from 2022 to 2030, we made estimates with five different COVID-19 prevalence assumptions: 0.5%, 1%, 5%, 10%, and 15% based on prevalence ranges reported on a global scale (see Table 1 for COVID-19 prevalence estimates in 2020 and 2021). The rates of hospitalization, ICU admission, and deaths from 2022-2030 estimates were set equal to those calculated for 2021. The all-age estimates were disaggregated by sex using the reported fraction of male and female cases of each country [23–25]. Additionally, the death estimates were disaggregated by age using data reported from each country. The additional non-ICU and ICU hospitalizations, and deaths related to OAO were obtained by comparing the estimates of group with BMI <25 kg/m2 to the estimates for those with OAO. Estimates from 2022 to 2030 were adjusted to include the effect of vaccination on hospitalization and death outcomes [26,27]. We simulated the occurrence of new variants that can escape vaccine protection. Thus, the vaccine effectiveness was set equal to the vaccine effectiveness against the Omicron variants for the projections from 2022 to 2030. In our model, effectiveness of the vaccine is not affected by BMI status, as a recent study has shown [28]. From 2022 to 2030, vaccination coverage was projected using non-linear generalized regression models based on country coverage reported in OurWorldInData.org [22]. The models were generalized additive models [29] with monthly vaccination coverage as the dependent variable and year as an independent variable from January 2021 to September 2022. We ran a model for first, second, and third (booster) vaccines.

**Table 1:** Additional inpatient cases/hospitalizations, ICU admissions and deaths (per 10,000 total population in country and as percentage of total COVID-19 outcomes) related to overweight and obesity in 2020 and 2021.

		Additional Hospitalizations Among Those with Overweight and Obesity		Additional ICU Admissions Among Those with Overweight and Obesity		Additional Deaths Among Those with Overweight and Obesity	
Country	COVID-19 prevalence	Per 10,000 population	Percent of total COVID inpatient cases	Per 10,000 population	Percent of total COVID ICU admissions	Per 10,000 population	Percent of total COVID deaths
**2020**							
Australia	0.29%	0.212	25.60%	0.197	53.01%	0.018	20.63%
Brazil	12.09%	8.780	21.81%	8.790	47.26%	1.631	17.73%
India	2.45%	0.438	5.90%	0.408	16.70%	0.048	4.42%
Mexico	4.13%	6.539	23.03%	10.981	42.30%	2.341	20.34%
Saudi Arabia	3.43%	7.347	24.61%	5.997	52.71%	0.425	23.77%
South Africa	6.00%	6.840	22.63%	8.010	46.83%	0.969	20.16%
Spain	14.55%	7.044	20.88%	7.138	48.12%	2.215	17.39%
Thailand	0.03%	0.026	13.34%	0.020	33.74%	0.001	12.70%
**2021**							
Australia	1.81%	0.815	26.06%	0.757	53.54%	0.071	21.36%
Brazil	17.54%	7.856	22.30%	7.881	48.04%	1.458	18.15%
India	4.87%	0.587	6.17%	0.549	17.41%	0.065	4.64%
Mexico	6.11%	5.715	23.53%	9.578	42.93%	2.046	20.79%
Saudi Arabia	1.29%	1.581	25.00%	1.293	53.25%	0.091	24.12%
South Africa	6.24%	6.088	23.28%	7.136	47.76%	0.864	20.78%
Spain	14.53%	3.961	19.43%	4.258	47.24%	1.247	16.21%
Thailand	1.45%	0.733	13.98%	0.560	35.17%	0.030	12.30%

### Cost calculations

Direct medical costs included the costs of additional hospitalizations and ICU admissions due to COVID-19 among the population with OAO. We calculated the inpatient/hospitalization cost by multiplying the average cost per COVID-19 case requiring hospitalization by the additional number of hospitalized cases. We calculated the costs of ICU treatment by multiplying the average cost per COVID-19 case requiring ICU treatment by the additional ICU admissions. These two cost components where then summed up to calculate the medical cost of treatments.

Premature mortality cost is the economic value of additional deaths due to COVID-19 among people with OAO. This was calculated as the OAO-related additional deaths (by age group and sex) from COVID-19 multiplied by the economic value they could have produced over their remaining years of life expectancy had they not died from COVID-19, considering background death rates. We represent the economic value of a life year as the country’s GDP per capita. All future costs are assigned to the year in which death occurred and discounted at a rate of 3% per year to obtain the net present value. The net present value of the economic cost of additional deaths for each sex and age cohort are summed to give the total economic cost of additional deaths among those with OAO.

Cost estimate projections from 2021 to 2030 were derived by extending the cost model used for 2020 to future years with projections of model parameters (Table C in S1 Table). Data on life expectancy and death rates were drawn from the UNPD database; projections for GDP per capita are from the IMF Economic Outlook. Future COVID-19 hospitalization, ICU admissions and mortality were modeled as described earlier.

### OAO prevalence projections

We employed a regression-based approach for projecting future OAO prevalence (Table D in S1 Table). We used the historical trend of country-level OAO prevalence estimates from 1975 to 2016 sourced from NCD Risk Collaboration group [30]. The data set provides estimates separately for males above 20 years old (referred to as men), females above 20 years of age (women), males below 20 years of age (boys) and females below 20 years of age (girls). Hence our projections are done for these four groups separately. Following the approach of Ward et al (2020) [31], we used multinomial regressions to predict the prevalence of BMI categories for each group. For adults, the BMI categories modelled were normal weight, overweight, moderate obesity, and severe obesity. For those below age 20, modeled BMI categories were underweight, normal weight, overweight, and obesity. This ensures that the sum of the prevalence of all categories does not exceed 100% and allows for estimation of nonlinear trends and movements of individuals between categories. We assume that trends in other variables such as urbanization and population changes (that change with time) are implicitly controlled for by having time as the predictor variable [11].

A full description of the methodology for direct medical costs, premature mortality cost, and OAO prevalence has been published previously [11,14].

### Economic impacts from future COVID prevalence scenarios

Given the variability in COVID-19 trajectory due to vaccine uptake and emergence of different strains, this study also included various future COVID-19 prevalence scenarios. Future additional COVID-19 hospitalizations, ICU admissions, and mortality were modeled as described earlier using scenarios of 0.5%, 1%, 5%, 10%, and 15% COVID-19 prevalence. The OAO-related cost of COVID-19 was calculated under each scenario.

In addition, to understand the potential impact of OAO prevalence reduction in the future, we modelled the associated costs for each COVID-19 prevalence scenario if OAO prevalence is kept constant at 2019 levels from 2020 to 2030 or if annual projected OAO prevalence is reduced by 50% through 2030. All analyses were conducted using R and Stata 15 [32,33].

## Results

### Additional hospitalizations, ICU admissions and deaths

**Fig 1:**
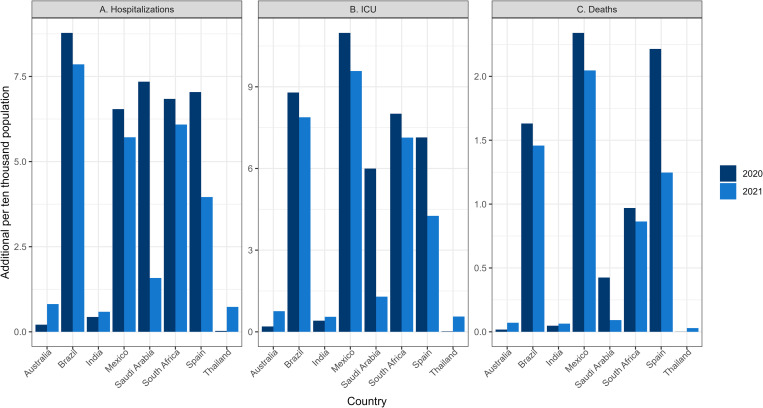
Additional deaths, hospitalizations, and ICU admissions from COVID-19 among population with overweight and obesity in 2020 and 2021 (per 10,000 population).

Table 1 shows the number of additional COVID-19 hospitalized cases, ICU admissions, and deaths affecting people with OAO (number of cases, admissions, and deaths that would not occur in the absence of OAO) as ‘per 10,000 population’ and as percentage of total COVID-19 outcomes in 2020 and 2021. In 2020, Brazil had the highest number of additional hospitalizations; Mexico had the highest number of additional ICU admissions and deaths per 10,000 population, while Thailand had the lowest of all three outcome measures (Fig 1). In 2021, Brazil had the highest number of additional hospitalizations, Mexico had the highest number of additional ICU admissions and deaths per 10,000 population, while India had the lowest additional hospitalizations and ICU admissions, and Thailand had the lowest number of additional deaths (Fig 1). This possibly reflects the influence of factors such as the COVID-19 prevalence, the strength of the public health system, and the level of development of health care infrastructure. On average, additional outcomes (hospitalizations, ICU admissions, and deaths) among those with OAO accounted for about 19.7%, 42.6% and 17.1% of COVID-19 hospitalizations, ICU admissions, and deaths, respectively, in 2020 across the eight countries. In 2021, additional outcomes (hospitalizations, ICU admissions, and deaths) among those with OAO accounted on average for about 20.0%, 43.2% and 17.3% of COVID-19 hospitalizations, ICU admissions and deaths, respectively, across the eight countries (Table 1).

### Treatment and premature mortality costs in 2020 and 2021

**Fig 2:**
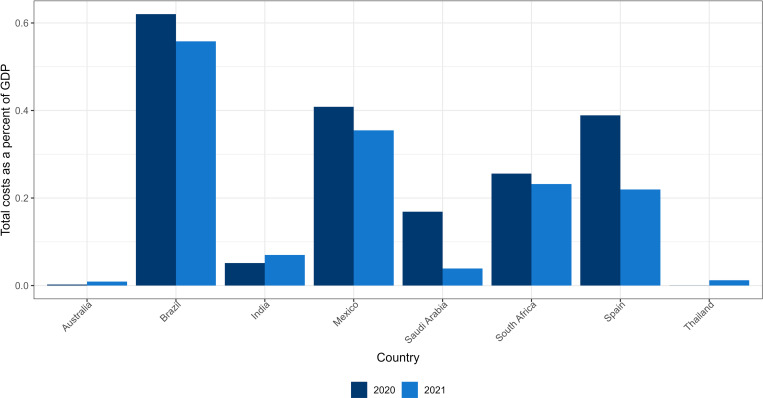
Total cost (treatment and premature mortality costs) among population with overweight and obesity as percent of GDP in 2020 and 2021.

Table 2 presents a summary of estimates of additional treatment costs and premature mortality costs due to COVID-19 among the population with OAO in the eight countries. In 2020, treatment costs ranged from US$0.64 million in Thailand to US$4.6 billion in Brazil while premature mortality costs ranged from US$1.48 million in Thailand to US$4.3 billion in Brazil. In 2021, treatment costs ranged from US$14.42 million in Australia to US$4.4 billion in Brazil while premature mortality costs ranged from US$42.12 million in Thailand to US$4.1 billion in Brazil. The treatment and premature mortality costs declined between 2020 and 2021 for countries where hospitalizations and deaths declined respectively (Brazil, Mexico, Saudi Arabia, South Africa, and Spain), while these costs increased for Australia, India, and Thailand, where COVID-19 prevalence, hospitalizations and deaths went up (S1–S3 Figs). As a percentage of total health expenditure, treatment costs range between 0.003% in Australia to 3.230% in Brazil in 2020 and between 0.01% in Australia to 2.98% in Brazil in 2021 (Table 2 and S4 Fig). Total costs (treatment and premature mortality costs) as a percent of GDP ranges between 0.0003% in Thailand to 0.62% in Brazil in 2020 and between 0.01% in Australia to 0.56% in Brazil in 2021(Table 2 and Fig 2). Total (treatment and premature mortality) costs are higher for females compared to males in Australia, Brazil, India, Mexico, South Africa, and Spain (S5–S7 Figs). This stems from an interplay of factors such as a different proportion of males and females in the OAO BMI categories, varying population sizes and life expectancies between sexes, and differences in the number and ages at which hospitalizations and deaths occur between the sexes, among others.

**Table 2:** Treatment costs and premature mortality costs in 2020 and 2021 (in constant 2020 USD).

Country	Treatment costs (millions)	Premature mortality costs (millions)	Total (millions)	Total cost per capita	Treatment cost as % of THE	Total cost as % of GDP
**2020**						
Australia	3.51	25.70	29.21	1.15	0.003%	0.002%
Brazil	4,640.70	4,342.00	8,982.70	42.26	3.230%	0.620%
India	1,028.49	344.45	1,372.94	0.99	0.959%	0.051%
Mexico	528.93	3,909.75	4,438.67	34.43	0.791%	0.408%
Saudi Arabia	641.42	544.86	1,186.29	34.08	1.355%	0.169%
South Africa	482.53	375.55	858.08	14.47	1.703%	0.256%
Spain	1,592.88	3,389.80	4,982.69	106.57	1.226%	0.389%
Thailand	0.64	1.48	2.12	0.03	0.003%	0.000%
**2021**						
Australia	14.42	112.14	126.56	4.91	0.011%	0.009%
Brazil	4,361.26	4,095.03	8,456.29	39.52	2.980%	0.558%
India	1,531.42	497.03	2,028.45	1.46	1.367%	0.070%
Mexico	485.78	3,554.37	4,040.15	31.02	0.707%	0.355%
Saudi Arabia	161.00	122.83	283.82	8.03	0.333%	0.039%
South Africa	464.12	351.92	816.04	13.59	1.596%	0.232%
Spain	959.53	1,998.91	2,958.44	63.29	0.729%	0.220%
Thailand	18.78	42.12	60.90	0.87	0.088%	0.012%

### Projected costs from scenarios

We estimated future costs from 2022 to 2030 using five potential COVID-19 prevalence scenarios (0.5%, 1%, 5%, 10% and 15%). S8–S10 Figs summarize the trajectory of total (treatment and premature mortality) costs with the different COVID-19 prevalence scenarios. Countries will have lower costs if future COVID-19 prevalence declines. The estimated additional deaths in all future scenarios from 2023 to 2030 are shown in S11 Fig. In all future COVID-19 scenarios, additional deaths among the population with OAO in 2030 could account for up to 22% of all COVID-19 deaths on average across the eight countries (S12 Fig).

Annual cost reduction in the potential scenario where OAO prevalence is kept constant at 2019 levels - i.e., achieving a flattening of the projected rise in future OAO prevalence - are shown in Table 3. Compared to total costs from baseline OAO prevalence projections, reductions across the different COVID-19 prevalence scenarios could range from 10.8%-12.5% of projected baseline total costs in Spain to 26.7-27.1% of projected baseline total costs in India between 2022 and 2030, approximately 17.4%-18.5% on average across the 8 countries. In the second scenario where projected baseline OAO prevalence till 2030 is reduced by 50%, average annual cost reduction - compared to total costs from baseline OAO prevalence projections - across the different COVID-19 prevalence scenarios could range from 35.9%-36.4% of projected baseline total costs in Saudi Arabia to 46.4-46.5% of projected baseline total costs in India between 2022 and 2030, approximately 40.8%-41.4% on average across the 8 countries.

**Table 3:** Average annual cost reductions (2022-2030) based on two overweight and obesity prevalence scenarios.

Country	Average annual cost reduction (in 2020 US$)					Percentage cost reduction (%)
	COVID-19 Prevalence 0.005	COVID-19 Prevalence 0.01	COVID-19 Prevalence 0.05	COVID-19 Prevalence 0.1	COVID-19 Prevalence 0.15	
**Scenario 1: If overweight and obesity prevalence is kept constant at 2019 levels through 2030**						
Australia	9,872,636	19,981,684	99,693,120	208,094,976	322,559,936	13.7%-15.5%
Brazil	124,830,416	250,587,216	1,251,572,864	2,521,659,392	3,769,757,952	17.1%-17.3%
India	119,242,232	241,576,896	1,194,648,320	2,381,070,592	3,587,185,408	26.7%-27.1%
Mexico	103,655,936	211,369,904	1,060,118,144	2,101,604,224	3,155,433,984	14.9%-15.1%
Saudi Arabia	27,842,962	54,676,952	283,773,600	572,672,064	846,308,608	11.7%-12.2%
South Africa	8,760,698	17,575,346	89,449,064	177,733,024	266,690,880	20.8%-21.2%
Spain	38,650,600	67,095,716	371,343,232	731,270,016	1,097,978,112	10.8%-12.5%
Thailand	12,952,786	29,775,932	146,022,320	297,109,632	441,636,864	23.4%-27.1%
**Scenario 2: If projected future overweight and obesity prevalence is reduced by 50% through 2030**						
Australia	25,105,804	49,948,908	258,927,744	524,182,944	779,737,088	38.2%-39.9%
Brazil	259,297,248	516,156,928	2,591,440,896	5,191,536,128	7,777,179,648	39.5%-39.6%
India	191,696,320	383,206,688	1,915,950,848	3,830,877,952	5,751,916,544	46.4%-46.5%
Mexico	289,973,376	580,876,992	2,906,056,704	5,803,076,096	8,703,380,480	43.6%-43.7%
Saudi Arabia	82,367,664	161,330,336	816,235,072	1,628,919,936	2,446,415,360	35.9%-36.4%
South Africa	14,954,819	30,419,814	151,465,440	305,169,344	456,712,896	38.0%-38.5%
Spain	109,754,952	217,003,568	1,102,484,480	2,202,667,008	3,291,579,392	39.3%-39.5%
Thailand	22,673,652	47,311,436	231,242,544	464,438,880	698,851,264	45.4%-46.7%

## Discussion

We find that additional COVID-19 outcomes associated with OAO accounted for a significant proportion of total COVID-19 hospitalizations, ICU admissions, and deaths – approximately 20% of hospitalizations, 43% of ICU admissions, and 17% of deaths in 2020 and 2021 on average across the eight countries included in the analysis (Table 2). Between 2022 and 2030, additional COVID-19 deaths due to OAO may account for up to a fifth of total COVID-19 deaths across the eight countries (S12 Fig). This is consistent with emerging evidence that has found an increased likelihood of death (up to a factor of 10) from COVID-19 in countries where more than half of the population are classified as overweight compared to those with less than half of the population classified as overweight [13]. In addition to the prevalence of OAO, the strength of the government’s COVID-19’s response across countries may impact the magnitude of OAO-related COVID-19 outcomes. *Foreign Policy*’s COVID-19 Global Response Index, which includes all the countries in this analysis except Thailand, reflects government actions for containing the virus, financial support for domestic economies, and fact-based communication among leaders [34]. The countries with the three worst scores in the Index were also the countries with the three highest rates of OAO-related deaths and ICU admissions (Brazil, Mexico, and Spain) [34]. For hospitalizations, two of the three lowest scoring countries had the highest OAO-related COVID-19 hospitalization rates (Brazil and Spain) [34].

We also find that the total costs (comprised of treatment costs and premature mortality costs) are not trivial. As a percentage of GDP, the total costs of COVID-19 related to OAO could conservatively have been up to 0.62% of GDP in 2020 and 0.56% in 2021 in Brazil. By comparison, a series of fiscal measures enacted by authorities in Brazil to mitigate the impact of COVID-19 added up to about 12% of GDP [35]. In addition, we find that total costs are higher for females than males across the majority of analyzed countries. This is driven by factors such as higher female population leading to higher treatment costs from a higher number of additional hospitalizations and ICU admissions, and higher life expectancy compared to males leading to higher premature mortality costs due to the greater number of potential years of life lost. Other factors include women’s higher representation among BMI categories for obesity and severe obesity in the population - consistent with the higher rates of obesity found among women than men overall - and disparities in health seeking behavior and access between sexes [36]. This implies that policy responses to address COVID-19 and OAO should take into consideration the gender dynamics and differences in outcomes.

Our projections to 2030 demonstrate the potential trajectory of health and economic impacts from OAO depending on various COVID-19 prevalence scenarios. The OAO-related impact will therefore vary for countries depending on the effectiveness of their COVID-19 response. Our projections are premised on population vaccine coverage percentages in 2022. Emerging evidence shows that vaccinations reduce the severity of cases and deaths [37,38]; hence, in the event of poorer vaccine coverage, our projected health and economic impacts could become even higher. We also demonstrate that if future OAO prevalence is halted at 2019 levels or reduced significantly, notable reductions in treatment and mortality costs, compared to baseline projections, could be achieved in the future. These all underscore the importance of increasing vaccine access as well as adequate prioritization and consideration of OAO reduction and prevention strategies to mitigate the reach and severity of infectious disease outbreaks.

### Limitations

This study has several limitations. First, our results are conservative as we do not include important cost components frequently included in cost-of-illness studies such as labor, education, and employment impacts of COVID-19. We were not able to include these costs due to very limited evidence on the differential impact of OAO on COVID-19 outcomes related to these measures. Hence, the full costs of COVID-19 that are associated with OAO will be substantially higher. In addition, there are obvious limitations inherent in the accuracy of the secondary data on hospitalizations and deaths including reporting errors and omissions which are transferred to our estimates. However, we use a single data source for consistency and comparability across countries. Also, the odds ratios used to calculate additional deaths are drawn from high-income countries [19–21] and therefore may not accurately apply to low- and middle-income countries. Despite these limitations, this paper provides initial evidence on the additional impacts of COVID-19 outcomes that result from OAO.

## Conclusion

The COVID-19 pandemic has brought the OAO challenge to the forefront of national health policy as a leading predictor of poor COVID-19 outcomes including mortality. We argue that the dichotomy between NCDs and infectious diseases is artificial and that the linkages across disease categories are increasingly evident. Hence, system-based approaches are needed to address escalations in comorbid conditions; and responses to the COVID-19 pandemic should also include broad-based approaches to reduce underlying risks, such as OAO. Investments in prevention and treatment of OAO would have the dual benefit of improving the health and wellbeing of populations while increasing the resiliency of health systems to future pandemics. Policymakers and public health professionals should therefore ensure optimal resource allocation and acceleration of the implementation of OAO policies across the prevention and treatment spectrum. The results of our analysis highlight the importance of recognizing OAO as a major risk factor for infectious diseases, as demonstrated by COVID-19. Our findings provide further empirical evidence in support of strengthened political commitment for national OAO interventions and prevention efforts to help increase resilience to public health emergencies in these and other countries.

## Supporting information

S1 Fig Treatment costs from additional hospitalizations and ICU admissions among population with overweight and obesity in 2020 and 2021 (in 2020 constant USD). (TIFF)

S2 Fig Premature mortality costs from additional deaths among population with overweight and obesity in 2020 and 2021 (in 2020 constant USD). (TIFF)

S3 Fig Total costs (treatment costs and premature mortality) among population with overweight and obesity in 2020 and 2021 (in 2020 constant USD). (TIFF)

S4 Fig Treatment costs from additional hospitalizations and ICU admissions among population with overweight and obesity as percent of total health expenditures (THE) in 2020 and 2021. (TIFF)

S5 Fig Treatment costs from additional hospitalizations and ICU admissions among population with overweight and obesity in 2020 (males and females). (TIFF)

S6 Fig Premature mortality costs from additional deaths among population with overweight and obesity in 2020 (males and females). (TIFF)

S7 Fig Total costs (treatment & premature mortality costs) among population with overweight and obesity in 2020 (males and females). (TIFF)

S8 Fig Projected total costs (treatment costs and premature mortality) among population with overweight and obesity 2022–2030 (in 2020 constant USD). (TIF)

S9 Fig Projected treatment costs from additional hospitalizations and ICU admissions among population with overweight and obesity, 2022–2030 (in 2020 constant USD) with different COVID prevalence scenarios (in 2020 constant USD). (TIF)

S10 Fig Projected premature mortality costs from additional deaths among population with overweight and obesity, 2022–2030 (in 2020 constant USD) with different COVID prevalence scenarios. (TIF)

S11 Fig Additional deaths among population with overweight and obesity (per 10,000 population) 2022–2030. (TIF)

S12 Fig Additional deaths among population with overweight and obesity as percentage of COVID-19 Deaths 2022–2030. (TIF)

S1 Table Supplementary Tables A–D. (DOCX)
