# Supplementary figures and images for: The economic impacts of COVID-19 hospitalizations, intensive care unit admissions, and deaths related to overweight and obesity
Source: PLOS Glob Public Health. 2025 Jun 4;5(6):e0001445. doi: 10.1371/journal.pgph.0001445 (PMC12136452; doi:10.1371/journal.pgph.0001445)

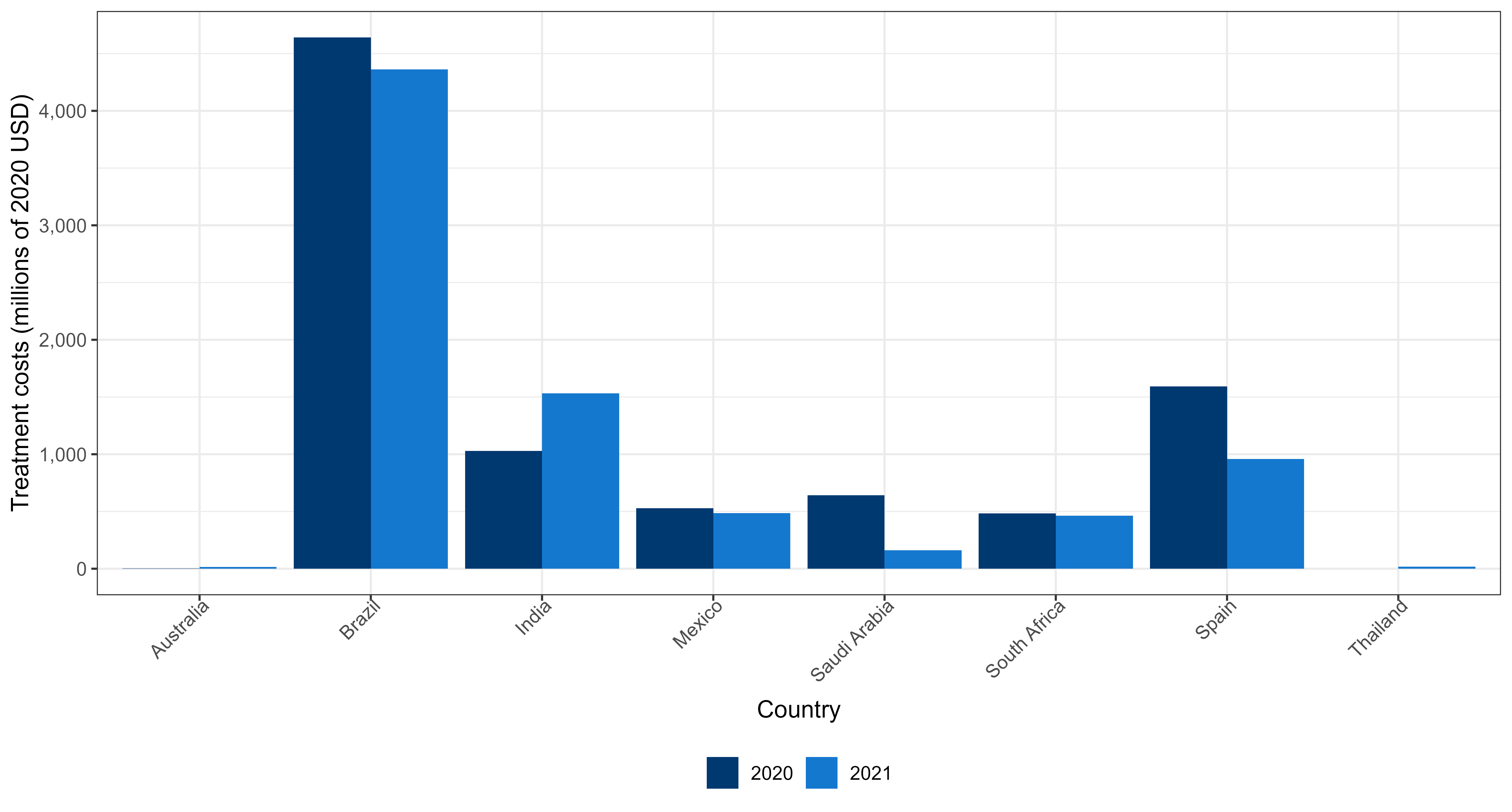

Supplement: S1 Fig — (TIFF) [file pgph.0001445.s001.tiff]

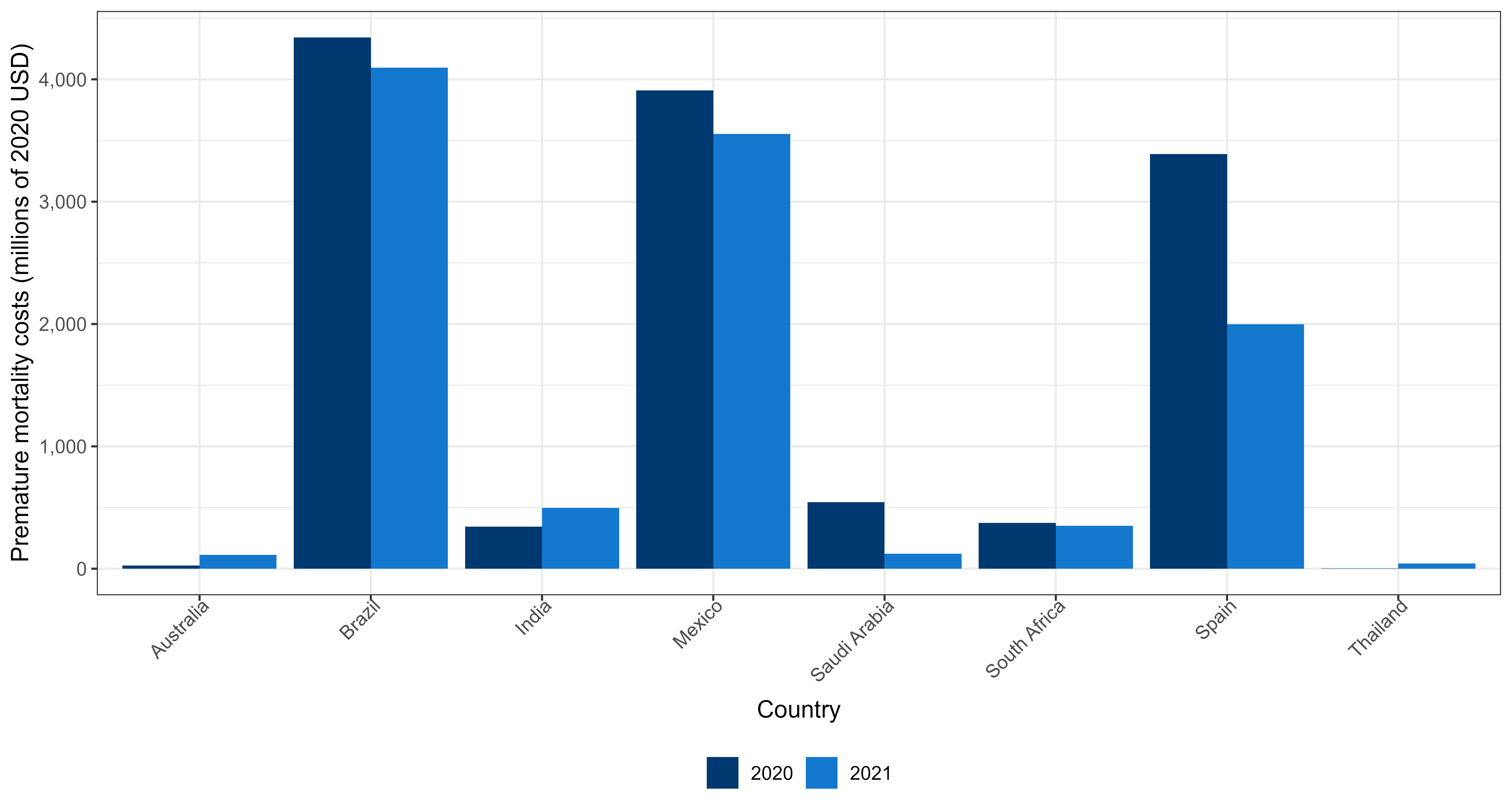

Supplement: S2 Fig — (TIFF) [file pgph.0001445.s002.tiff]

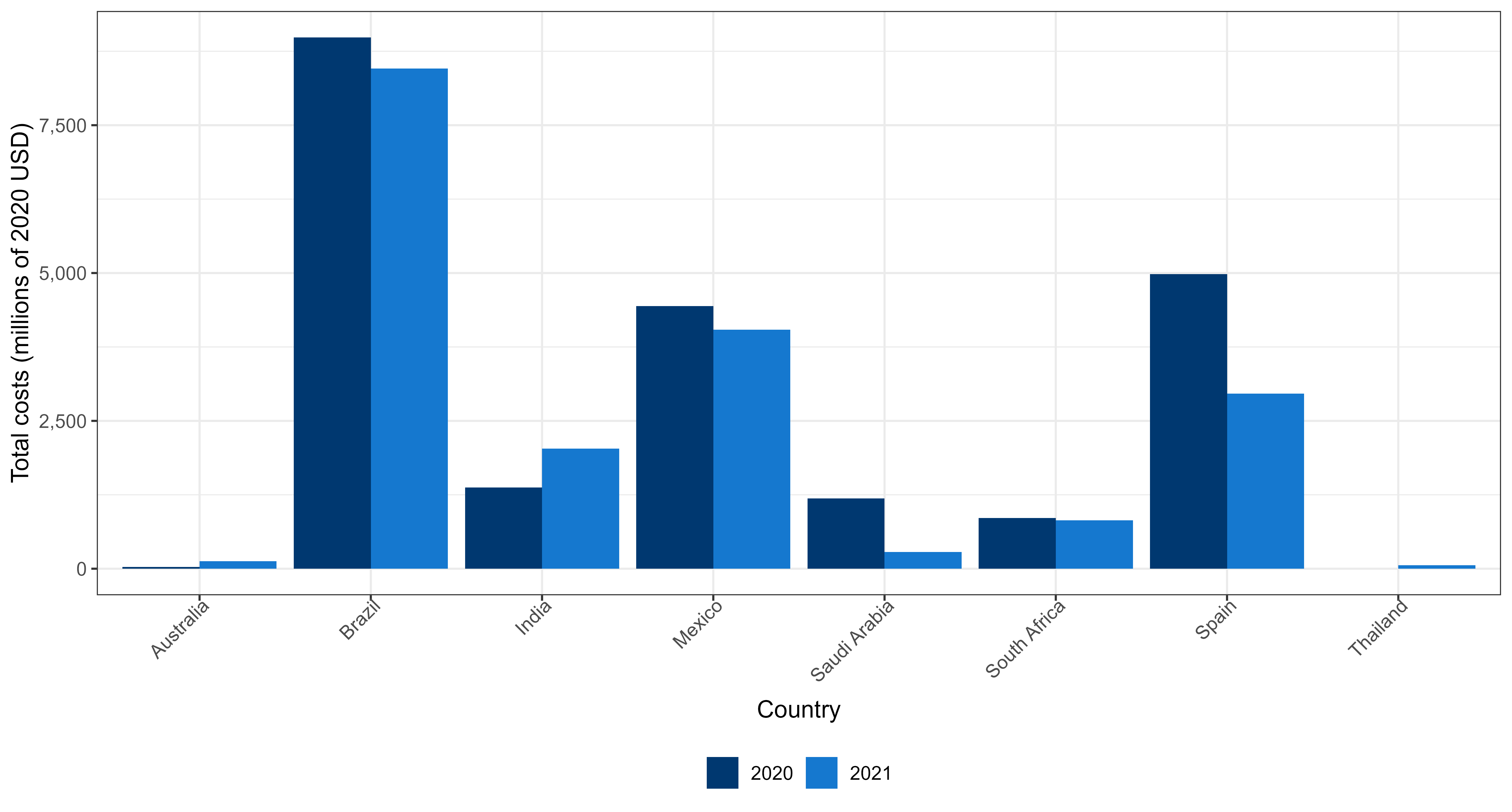

Supplement: S3 Fig — (TIFF) [file pgph.0001445.s003.tiff]

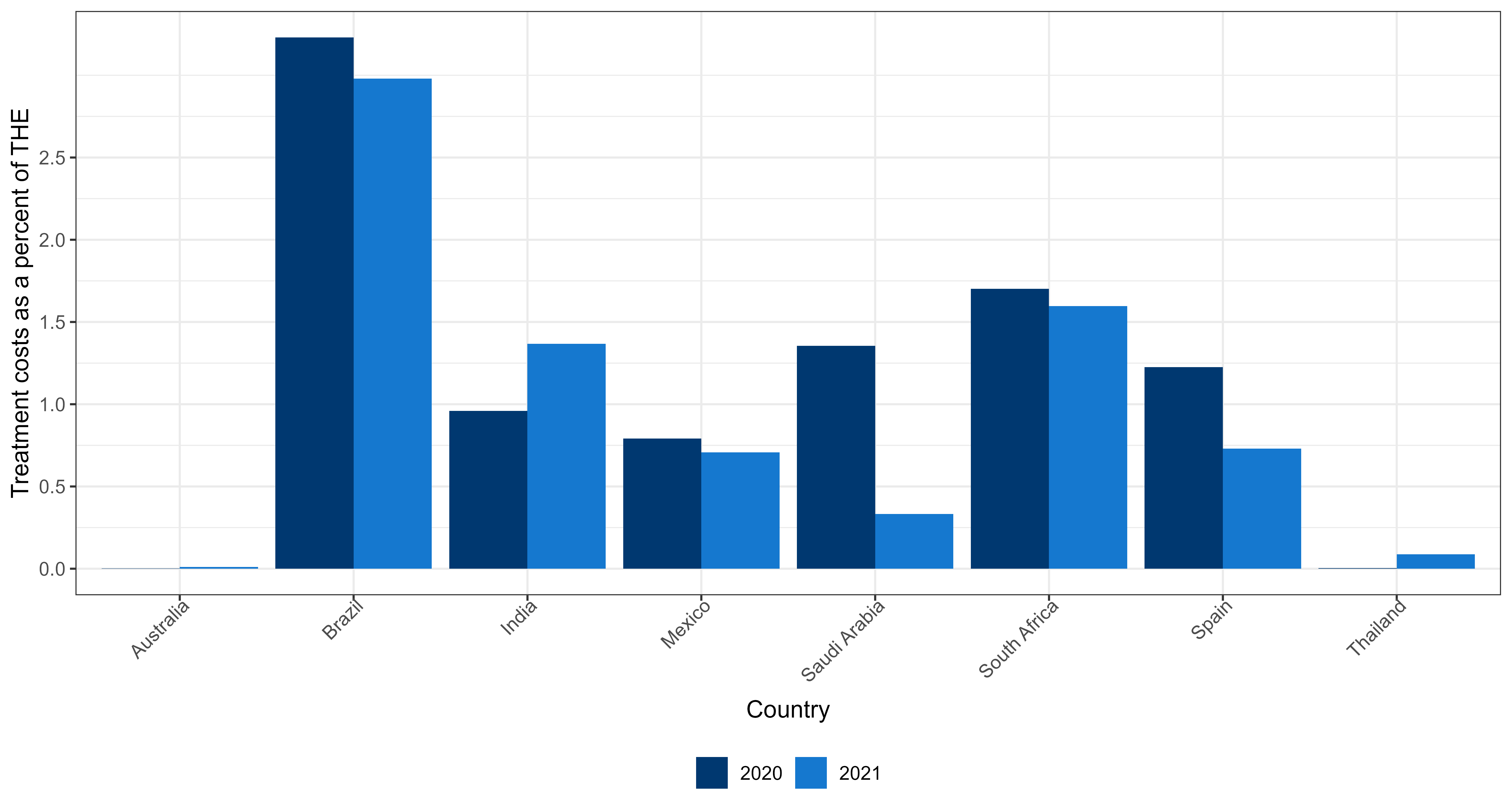

Supplement: S4 Fig — (TIFF) [file pgph.0001445.s004.tiff]

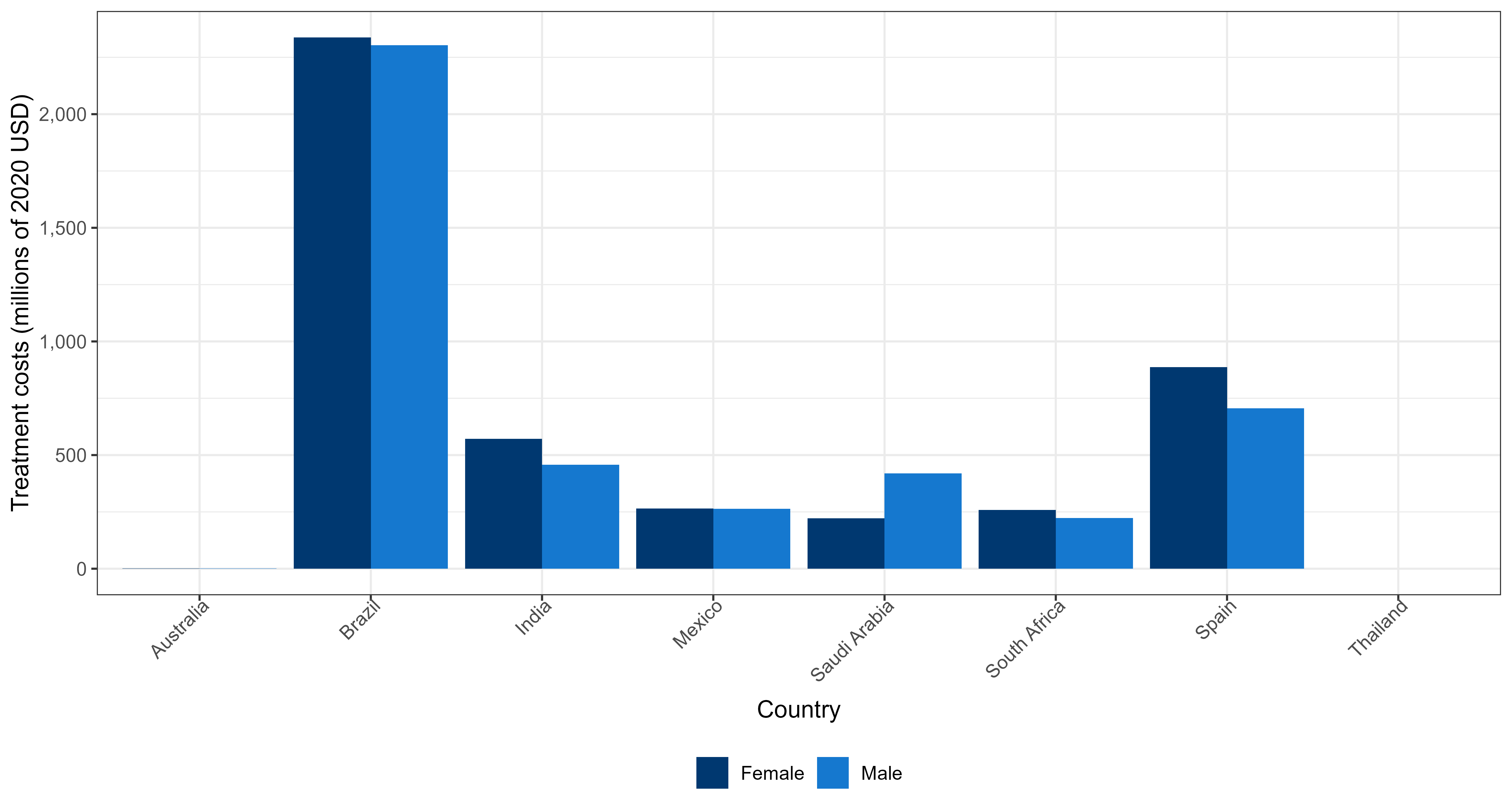

Supplement: S5 Fig — (TIFF) [file pgph.0001445.s005.tiff]

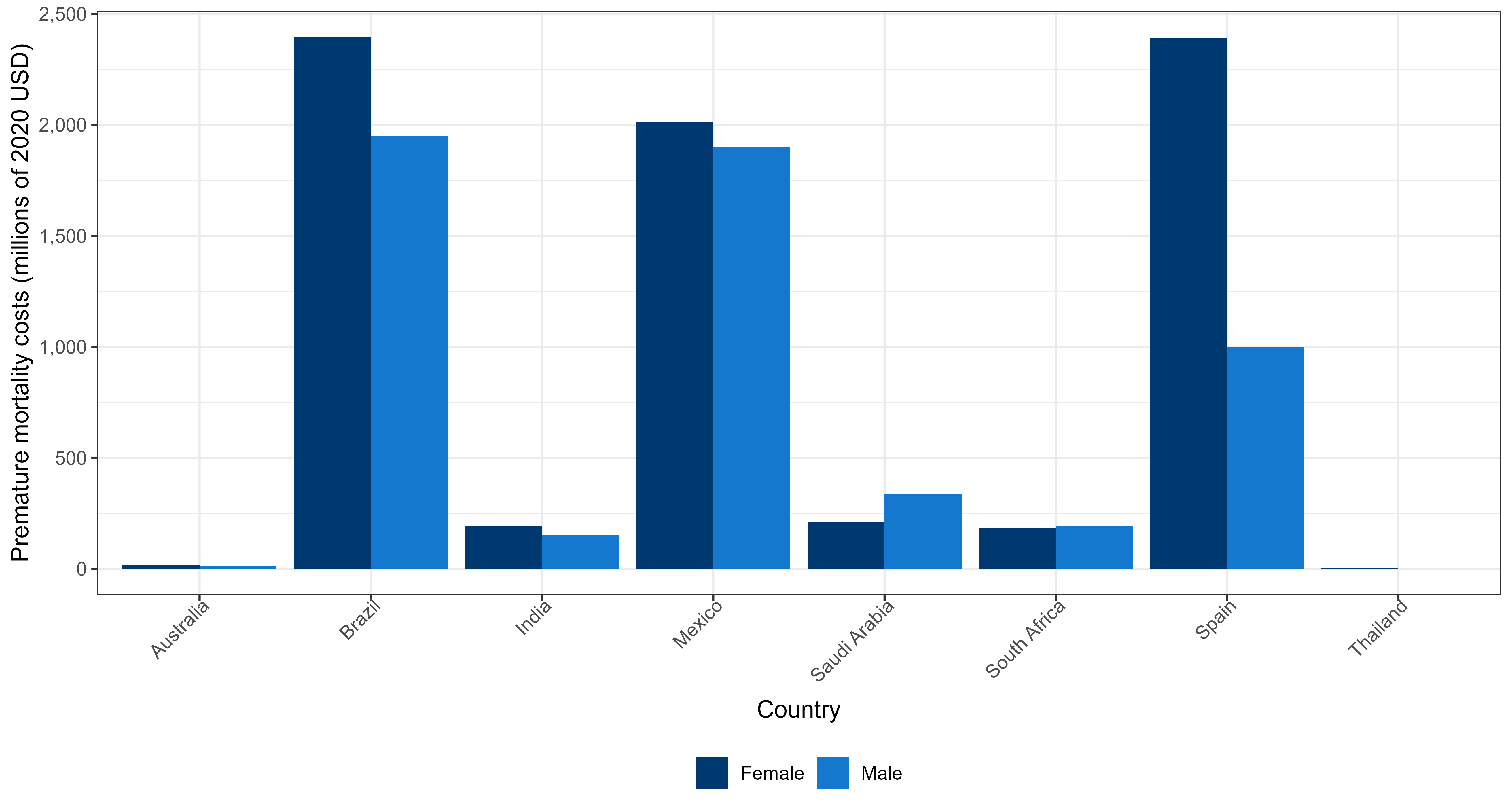

Supplement: S6 Fig — (TIFF) [file pgph.0001445.s006.tiff]

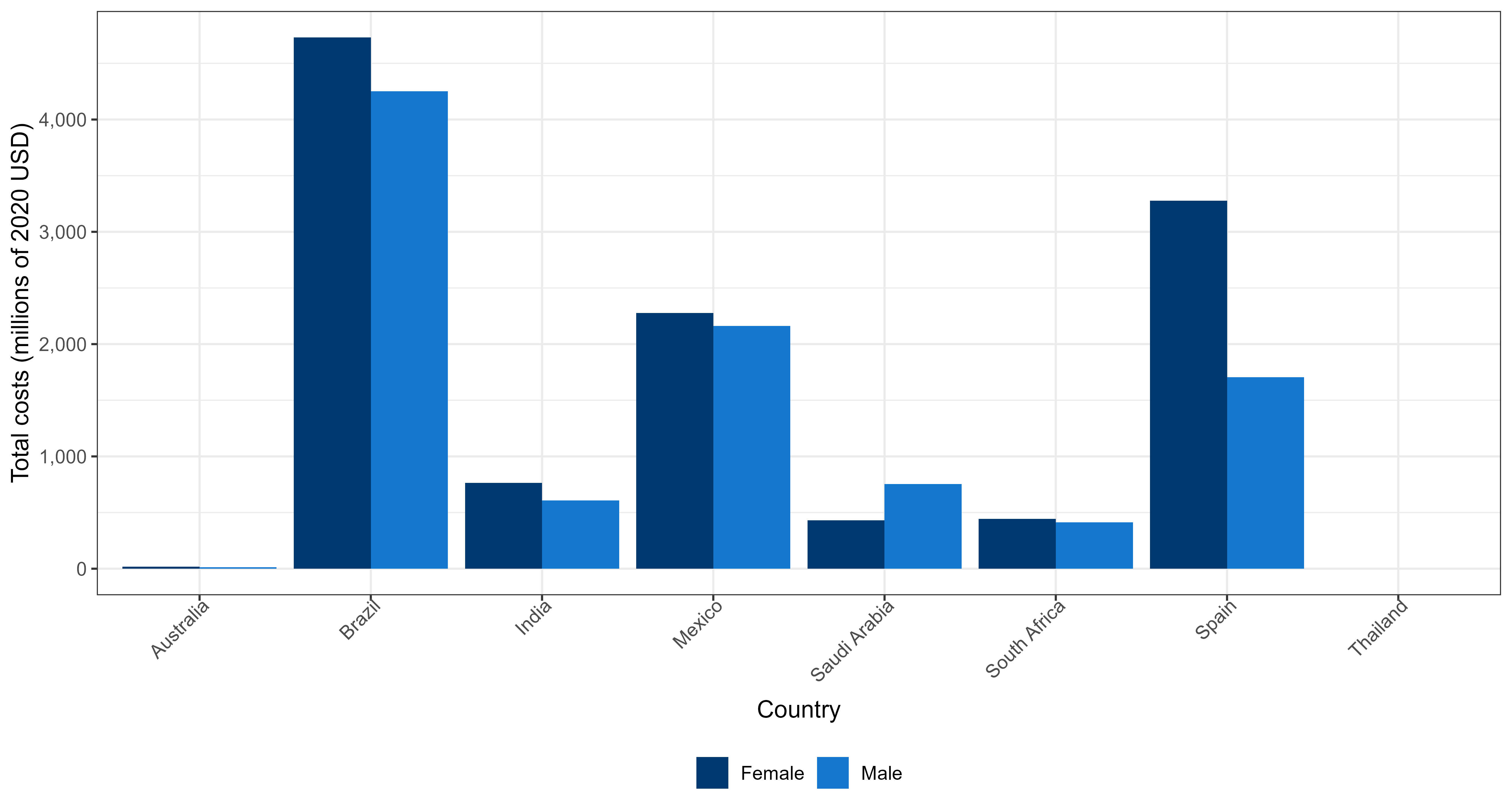

Supplement: S7 Fig — (TIFF) [file pgph.0001445.s007.tiff]

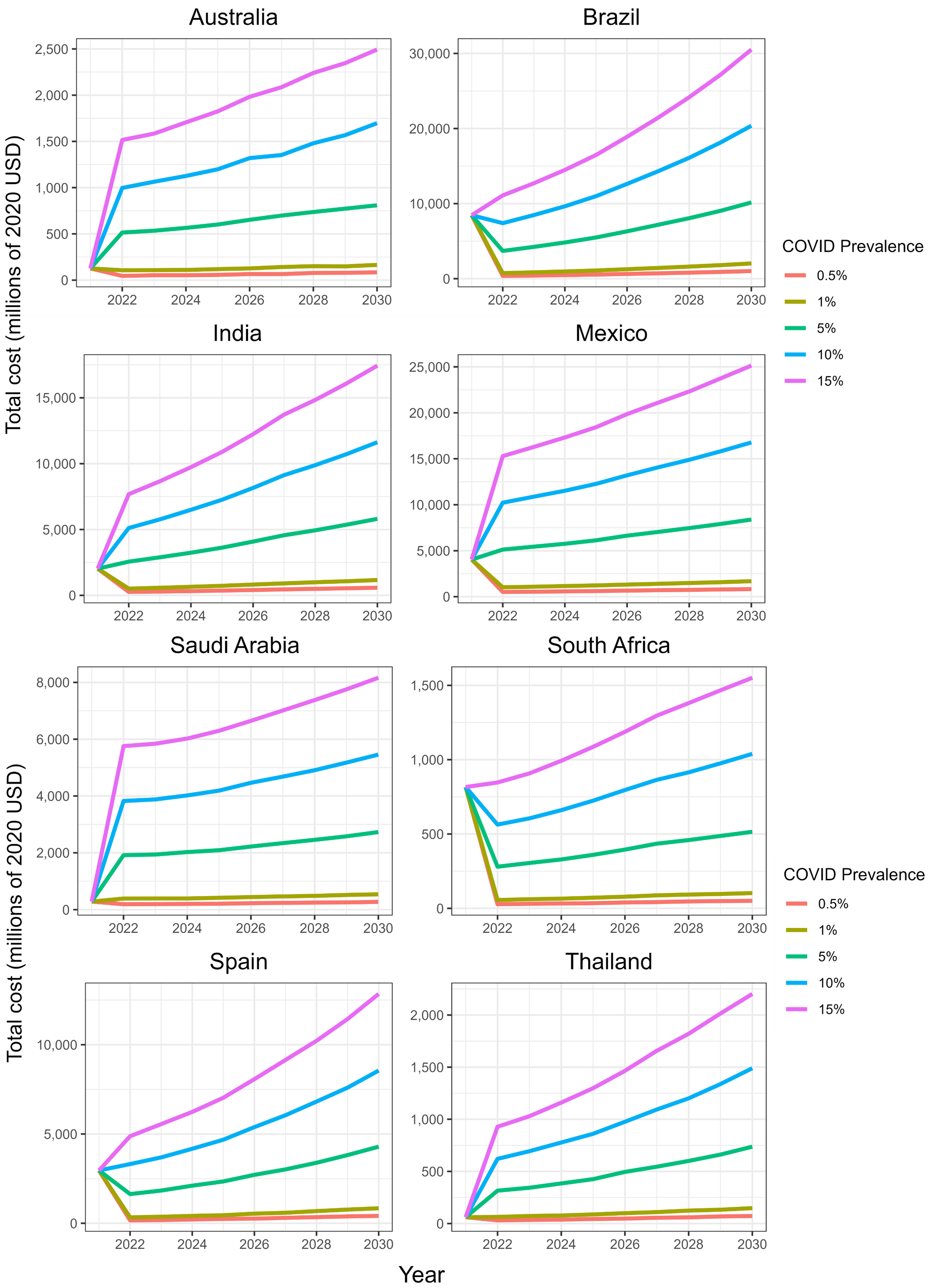

Supplement: S8 Fig — (TIF) [file pgph.0001445.s008.tif]

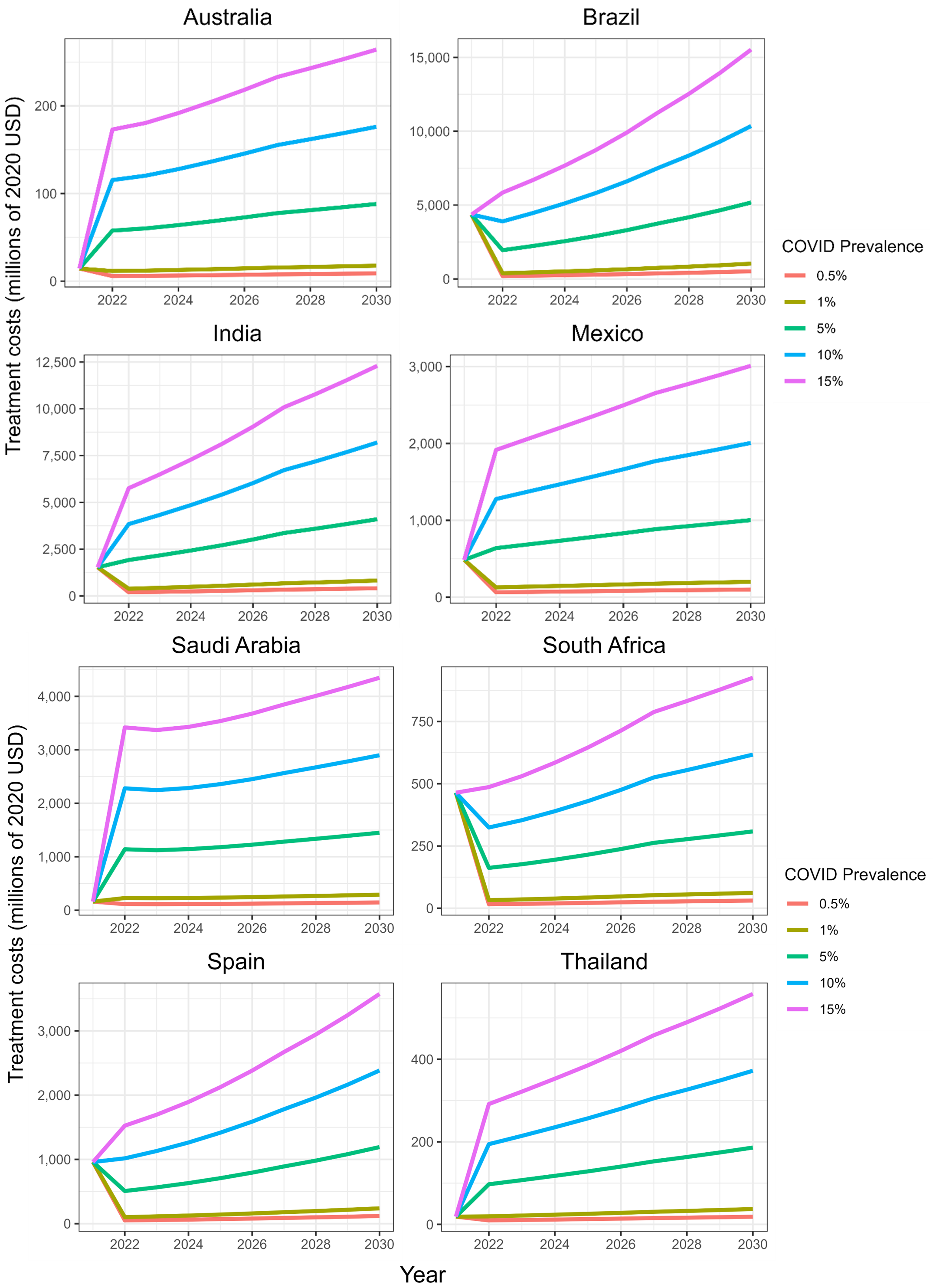

Supplement: S9 Fig — (TIF) [file pgph.0001445.s009.tif]

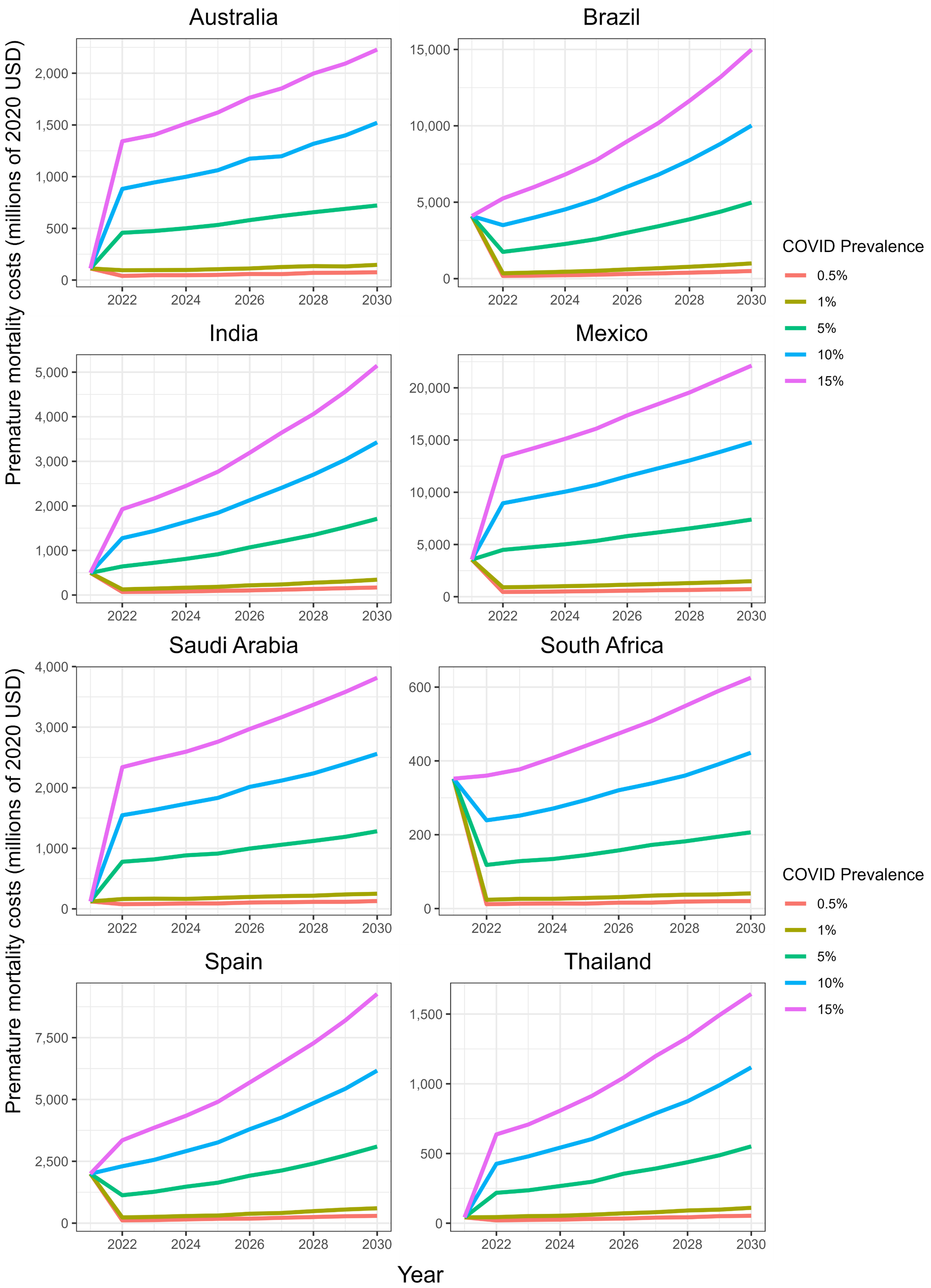

Supplement: S10 Fig — (TIF) [file pgph.0001445.s010.tif]

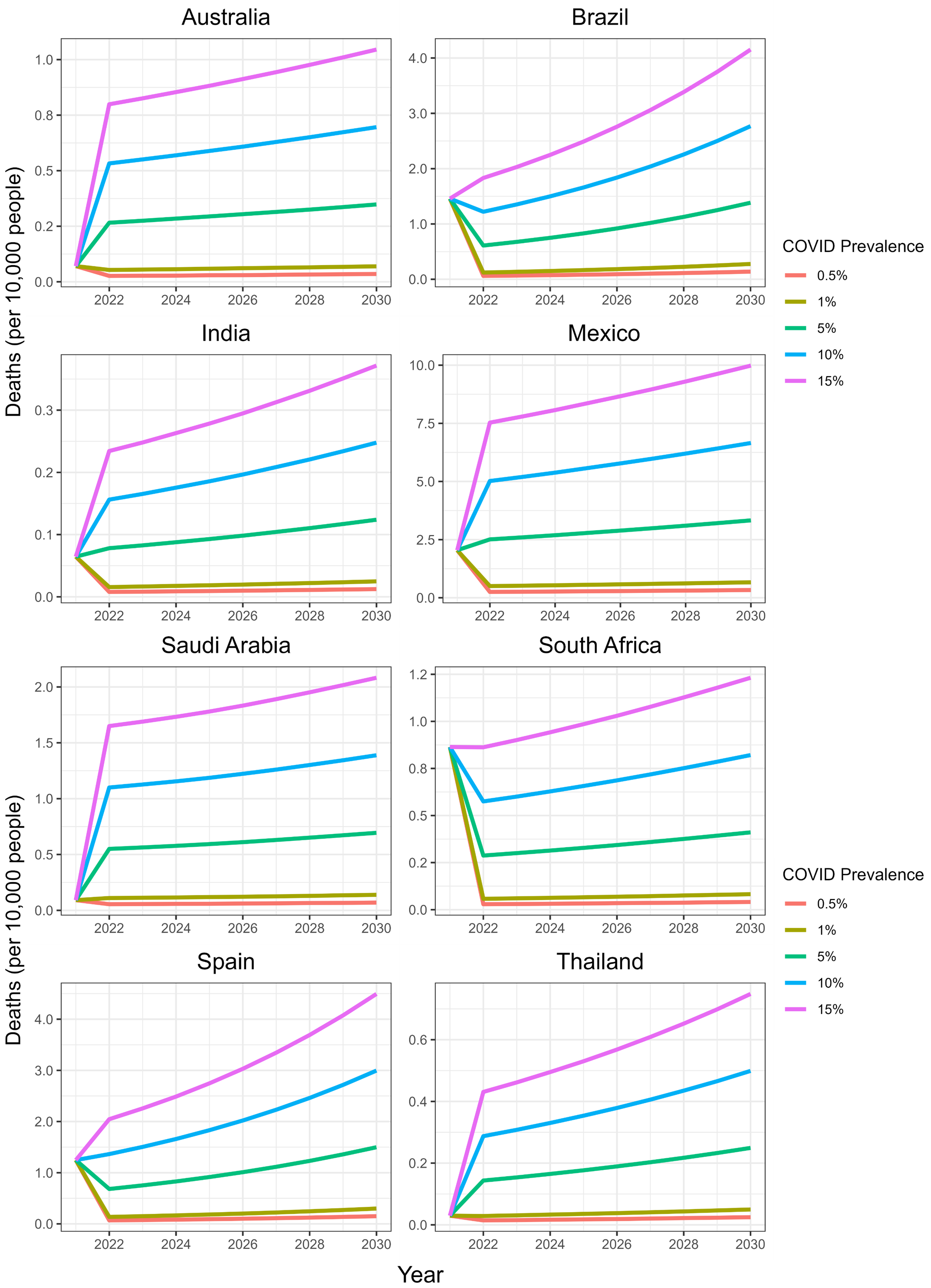

Supplement: S11 Fig — (TIF) [file pgph.0001445.s011.tif]

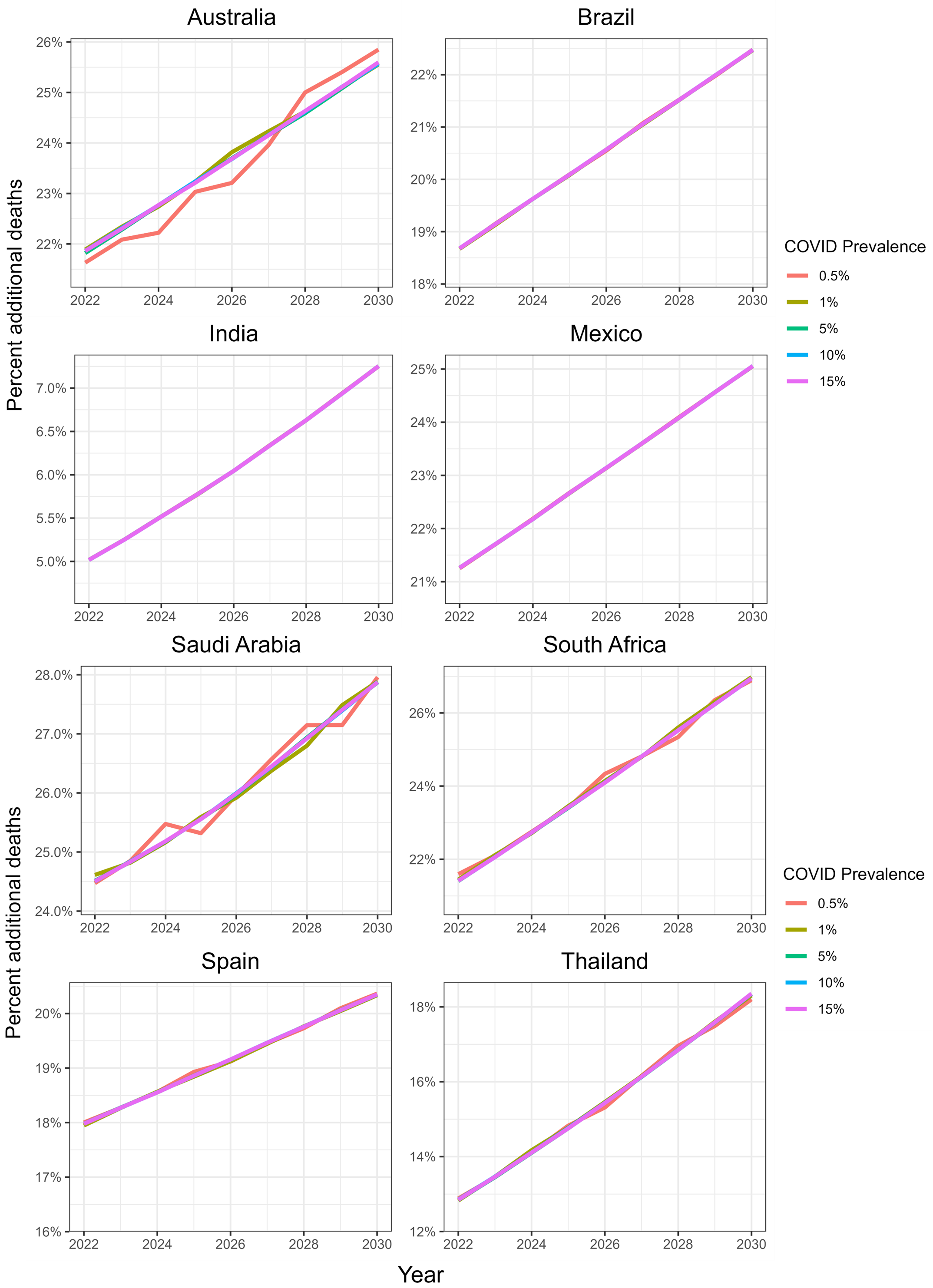

Supplement: S12 Fig — (TIF) [file pgph.0001445.s012.tif]
