# Supplementary material for: The economic impacts of COVID-19 hospitalizations, intensive care unit admissions, and deaths related to overweight and obesity
Source: PLOS Glob Public Health. 2025 Jun 4;5(6):e0001445. doi: 10.1371/journal.pgph.0001445 (PMC12136452; doi:10.1371/journal.pgph.0001445)
Supplement: S1 Table — (DOCX) [file pgph.0001445.s013.docx]

**Full title: The economic impacts of COVID-19 hospitalizations, intensive care unit admissions, and deaths related to overweight and obesity**

**Short title: The economic impacts of COVID-19 related to overweight and obesity**

Adeyemi Okunogbe^1*^, Donal Bisanzio^2^, Garrison Spencer^2^, Shradha Chhabria^3^, Jaynaide Powis^4^, Rachel Nugent^2^

^1^ RAND Corporation, Arlington, Virginia, United States of America

^2^ RTI International, Research Triangle Park, North Carolina, United States of America

^3^ Departments of Internal Medicine and Pediatrics, University Hospitals Cleveland Medical Center and Rainbow Babies and Children’s Hospital, Cleveland, Ohio, United States of America

^4^World Obesity Federation, London, EC1N 2SW, United Kingdom

* Corresponding author

Email: okunogbe@rand.org

**Table A in S1 Tables. Model summary: Parameters and data sources for obesity-related cost of COVID-19.**

| **Parameter** | **Used to Calculate** | **Data Source** |
| --- | --- | --- |
| Cost of inpatient/hospitalized care per COVID-19 case | Direct medical cost: Inpatient/hospitalization care | Peer-reviewed literature[1–6] |
| Number of additional OAO-related inpatient COVID-19 cases | Direct medical cost: Inpatient/hospitalization care | IHME Database and OurWorldInData.org [7] |
| Cost of ICU care per COVID-19 case | Direct Medical cost: ICU care | Peer-reviewed literature[1–6] |
| Number of additional OAO-related ICU COVID-19 cases | Direct Medical cost: ICU care | IHME Database and OurWorldInData.org [7] |
| Additional OAO-related COVID-19 mortality: No. of additional deaths among population with OAO compared with healthy weight population | Indirect Cost: Premature Mortality | IHME Database and OurWorldInData.org [7] |
| Overweight & Obesity prevalence | Indirect Cost: Premature Mortality | NCD Risk Factor Collaboration (NCD-RisC)[8] |
| Life expectancy | Indirect Cost: Premature Mortality | United Nations Population Division (UNPD)[9] |
| Background death rates | Indirect Cost: Premature Mortality | United Nations Population Division (UNPD)[9] |
| Annual gross domestic product (GDP) per capita | Indirect Cost: Premature Mortality | IMF World Economic Outlook |

**Table B in S1 Tables. Results from scoping review of overweight and obesity impacts on COVID-19 outcomes.**

| **Study Title** | **Outcome** | **Results** |
| --- | --- | --- |
| Yang, Hu, and Zhu (2021)[10] | Risk of Severe COVID-19 Disease | Mean Difference in BMI among those with severe COVID-19 disease: WMD=2.67, 95% CI[1.52, 3.82] & OR of patients with obesity developing severe COVID disease: 0OR=2.31, 95% CI[1.3, 4.12] |
| Huang et al. (2020)[11] | Risk of COVID-19 Hospitalization | Univariate analysis: OR of severe COVID-19 with higher BMI = 1.76, 95% CI (1.21, 2.56), p=0.003 Multivariate analysis: OR=2.36 (1.37, 4.07), p=0.002 Mean difference in adipose tissue: SMD=0.49 (0.11, 0.87), p=0.155 |
|  | Risk of ICU Admission | Univariate analysis: OR = 1.67, 95% CI (1.26, 2.21), p<0.001 Multivariate analysis: OR=2.32 (1.38, 3.90), p=0.001 Mean difference in adipose tissue: SMD=0.57 (0.33, 0.81), p=0.202 |
|  | Risk of Mechanical Ventilation | Univariate analysis: OR=2.19, 95%CI (1.56, 3.07), p<0.001 Multivariate analysis: OR=2.63 (1.32, 5.25), p=0.006 Mean difference in adipose tissue: SMD=0.37 (0.03, 0.71), p=0.468 |
|  | Risk of Mortality | Univariate analysis: OR=1.37, 95%CI (1.06, 1.75) Multivariate analysis: OR=1.49, 95%CI (1.20, 1.85), p=0.003 |
| Tamara and Tahapary (2020)[12] | Risk of Severe COVID-19 Disease | OR=1.30 (1.09, 1.54), p=0.003 for BMI≥25 |
|  | Risk of COVID-19 Hospitalization | OR=2.0 (1.6, 2.6), p<0.0001 compared to normal weight and OR=2.2 (1.7, 2.9), p<0.006 compared to overweight |
|  | Risk of ICU Admission | OR=1.8 (1.2, 2.7), p<0.0006 compared to normal weight and OR=3.6 (2.5, 5.3) p<0.0001 compared to overweight |
|  | Risk of Mechanical Ventilation | OR=7.36 (1.63, 33.14), p=0.021 for BMI≥35 |
| Földi et al. (2020)[13] | Risk of ICU Admission | OR=1.21 (1.002, 1.46) |
|  | Risk of Mechanical Ventilation | OR=2.05 (1.16, 3.64) |
| Malik et al. (2020)[14] | Risk of COVID-19 Diagnosis | Prevalence of COVID-19 infection among those with BMI>25 = 0.34 (0.23-0.44), vs prevalence=0.60 (0.34-0.86) among those with BMI<25 |
| Chang TH, Chou CC, and Chang LY (2020)[15] | Risk of Severe COVID-19 Disease | Mean BMI Difference in those with severe disease (vs mild/mod): MD=1.6 (0.8-2.4), p=0.0002 |
|  | Risk of COVID-19 Hospitalization | OR=1.4 (1.3-1.60), p<0.00001 |
|  | Risk of Mechanical Ventilation | BMI MD=4.1 (2.1-6.1), p<0.0001 and OR of IMV among those with obesity =2.0 (1.4-2.9), p<0.0001 |
| de Carvalho Sales-Peres et al. (2020)[16] | Other | Severe complications event rate = 56.2% (35.3-75.1) among patient with obesity |
| Soeroto et al. (2020)[17] | Other | Mean BMI Difference in patients with composite poor outcome = 1.12 (0.67-1.57), p<0.001 and OR for composite poor outcome in patients with obesity = 1.78 (1.25-2.54), p<0.001 |
| Seidu et al (2021)[18] | Risk of Severe COVID-19 Disease | RR=2.35 (1.43-3.86) for BMI≥25 (vs BMI<25) and RR=3.76 (1.97-7.16) for BMI>35 (vs BMI<25) |
|  | Risk of Mortality | RR=3.52 (1.32-9.42) for BMI≥25 (vs BMI<25) |
| Malik et al (2021)[19] | Other | OR of poor outcomes = 1.88 (1.25-2.80), p=0.002 |
| Chu et al. (2020)[20] | Risk of Severe COVID-19 Disease | Mean BMI Difference among those with severe disease: MD=2.48 (2.00-2.96), OR=4.17 (2.32-7.48) for risk of severe disease among patients with obesity |
|  | Risk of ICU Admission | OR=1.57 (1.18-2.09) |
|  | Risk of Mechanical Ventilation | OR=2.13 (1.10-4.14) |
|  | Risk of Mortality | No increased risk found here, OR=0.89 (0.32-2.51) |
|  | Other | OR for disease progression (distinct from severity) = 1.41 (1.26-1.58) and composite OR for poor outcomes (all previous measures) = 1.683 (1.408-2.011) |
| Yang et al, (2021)[21] | Risk of COVID-19 Diagnosis | OR=1.50 (1.37-1.63) |
|  | Risk of COVID-19 Hospitalization | OR=1.54 (1.33-1.78) |
|  | Risk of ICU Admission | OR=1.48 (1.24-1.77) |
|  | Risk of Mechanical Ventilation | OR=1.47 (1.31-1.65) |
|  | Risk of Mortality | OR=1.14 (1.04-1.26) |
| Földi et al. (2021)[22] | Risk of ICU Admission | Higher mean difference in visceral adipose tissue mass: SMD= 0.46 (0.20-0.71), p<0.001 |
|  | Risk of Mechanical Ventilation | Higher mean difference in visceral adipose tissue mass: SMD= 0.38 (0.05-0.71), p=0.026 |
| Zhao et al. (2020)[23] | Risk of Severe COVID-19 Disease | OR=2.07 (1.53-2.81) for severe disease or ICU admission |
|  | Risk of ICU Admission | OR=2.07 (1.53-2.81) for severe disease or ICU admission |
|  | Risk of Mortality | OR=1.57 (0.85-2.90), NOT signficant |
|  | Other | OR=3.76 (2.67-5.28) for the risk of severity and mortality among those with severe obesity |
| Hoong et al. (2021)[24] | Risk of Severe COVID-19 Disease | OR=2.26 (1.47-3.48), p<0.001 |
|  | Risk of Mortality | OR=1.51 (1.13-2.21), p=0.006 |
|  | Other | adjusted OR of unfavorable outcomes = 2.02 (1.41-2.89), p<0.001 |
| Shi et al. (2021)[25] | Risk of Severe COVID-19 Disease | OR = 2.47 (2.00, 3.04), p = 0.591; NOT significant [obese vs non-obese] |
|  | Risk of ICU Admission | OR = 1.66 (1.10, 2.50), p = 0.712; NOT significant [obese vs non-obese] |
|  | Risk of Mortality | OR = 1.89 (0.60, 5.91); NOT significant [obese vs non-obese] |
| Cai et al. (2021)[26] | Risk of COVID-19 Diagnosis | OR = 2.73 (1.53, 4.87) [obese vs non-obese] |
|  | Risk of Severe COVID-19 Disease | OR=3.81 (1.97,7.35) [obese vs non-obese] |
|  | Risk of COVID-19 Hospitalization | OR = 1.72 (1.55, 1.92) [obese vs non-obese] |
|  | Risk of ICU Admission | OR =2.25 (1.55, 3.27) [obese vs non-obese] |
|  | Risk of Mechanical Ventilation | OR = 1.66. (1.42,1.94) [obese vs non-obese] |
|  | Risk of Mortality | OR = 1..61 (1.29,2.01) [obese vs non-obese] |
| Zhou et al. (2020)[27] | Risk of Severe COVID-19 Disease | OR = 1.72 (1.04-2.85) [obese vs non-obese] |
| Groah et al. (2022)[28] | Risk of COVID-19 Diagnosis | Mean BMI Difference: MD = 4.0, p<0.01 |
| Harrison et al. (2021)[29] | Risk of Mortality | OR = 2.18 (1.10-4.34) [obese vs non-obese] |
| Popkin et al. (2020)[30] | Risk of COVID-19 Diagnosis | OR = 1.46 (1.30-1.65), p<0.0001 [obese vs non-obese] |
|  | Risk of COVID-19 Hospitalization | OR = 2.13 (1.74-2.60), p<0.0001 [obese vs non-obese] |
|  | Risk of ICU Admission | OR = 1.74 (1.46-2.08) [obese vs non-obese] |
|  | Risk of Mortality | OR = 1.48 (1.22-1.80), p<0.001 [obese vs non-obese] |
| Dessie and Zewotir (2021)[31] | Risk of Mortality | pOR = 1.34 (1.17-1.52) pHR = 1.50 (1.26-1.75) [obese vs non-obese] |
| Das et al. (2020)[32] | Other | Adverse outcome (death or ICU admission or needing IMV or hospitalization) OR = 2.81 (2.33-3.40) [obese vs non-obese] |
| Aghili et al (2021)[33] | Risk of ICU Admission | BMI >=30 OR = 1.189 (0.955, 1.424) [obese vs non-obese] |
|  | Risk of Mechanical Ventilation | BMI >= 30 OR = 2.049 (1.420,2.678) [obese vs non-obese] |
|  | Risk of Mortality | BMI >=30 OR = 1.35 (1.241, 1.459) [obese vs non-obese] |
|  | Other | Poor outcome (BMI >= 30) OR = 1.297 (1.178,1.416), p<0.001 [obese vs non-obese] |
| de Frel et al. (2020)[34] | Risk of Severe COVID-19 Disease | Each unit increase in BMI was associated with a 12% increase in the risk of severe disease |
| Hu and Wang (2021)[35] | Risk of Severe COVID-19 Disease | OR = 2.380 (0.995,5.695) [obese vs non-obese] |
|  | Risk of ICU Admission | OR = 1.267 (0.706,2.276) [obese vs non-obese] |
| Taylor et al. (2021)[36] | Risk of ICU Admission | BMI OR = 28.9 (28.2,29.7) |
| Rico-Martín et al. (2021)[37] | Risk of Mortality | OR = 2.13 (1.30,3.49) [obese vs non-obese] |
| Kirstensen et al. (2022)[38] | Risk of Severe COVID-19 Disease | BMI >=30 vs. BMI < 30 OR = (1.62, 4.31) |
|  | Risk of COVID-19 Hospitalization | BMI >= 30 vs. BMI < 30 OR = (1.40, 2.45) |
|  | Risk of ICU Admission | BMI >= 30 vs. BMI < 30 OR = (1.30,2.32) |
|  | Risk of Mechanical Ventilation | BMI >= 30 vs. BMI < 30 (1.47,2.63) |
| Booth et al. (2021)[39] | Risk of Severe COVID-19 Disease | BMI > 40 vs. BMI <= 40; OR = 2.57 (1.31,5.05) |
| Chowdhury et al. (2021)[40] | Risk of Severe COVID-19 Disease | OR = 2.09 (fixed effect) and 2.41 (random effect) [obese vs. non-obese patients] OR = 1.31 (pooled risk) [overweight vs BMI <25 patients] |
| Sahu et al. (2021)[41] | Risk of Severe COVID-19 Disease | OR = 1.89 (1.44,2.46) [BMI >= 30 vs. BMI <30] |
| Poly et al. (2021)[42] | Risk of Mortality | RRadjust: 1.42 (1.24-1.63) [obesity vs non-obese] Class 1 obesity: 1.27 (1.05-1.54), p=0.01 [class 1 vs BMI < 30] Class 2 obesity: 1.56 (1.11-2.19), p <0.01 [class 2 vs BMI < 35] Class 3 obesity: 1.19 (1.07-1.32), p=0.001 [class 3 vs BMI <40] |
| Xu, Mao, and Chen (2020)[43] | Risk of Severe COVID-19 Disease | BMI; OR = 3.38 (0.07, 6.69) [continuous] |
| Li et al. (2021)[44] | Risk of Mortality | OR = 1.59 (1.02,2.48) [obese vs. non-obese] |
| Du et al. (2020)[45] | Risk of COVID-19 Diagnosis | Patients with BMI >= 30 OR = 2.35 (1.64,3.38), p <0.001 (compared to patients with BMI <30) [obese vs. non-obese] |
|  | Risk of Severe COVID-19 Disease | Patients with BMI > 30 and age >60 years OR =3.11 (1.73,5.61), p<0.001 Patients with a BMI >= 30 had an OR = 2.35 (1.64, 3.38), p<0.001 compared with patients with a BMI < 30 [obese vs. non-obese] |
|  | Risk of Mortality | Patients with obesity and age >60 and BMI >30 OR = 3.93 (2.18,7.09), p<0.001 Random-effects dose-response meta-analysis showed a linear association between BMI and both critical COVID-19 and mortaility. The risk of critical COVID-19 and mortaility increased by 9% (OR = 1.09 (1.04,1.14), p<0.001) and 6% (OR = 1.06 (1.02,1.1), p = 0.002) for each 1 unit increase in BMI [obese vs. non-obese] |
| Geng et al. (2021)[46] | Risk of Severe COVID-19 Disease | OR = 2.63 (1.70-4.07), p = 0.000 [obese vs. non-obese] |
|  | Risk of ICU Admission | OR = 1.86 (1.49-2.31), p = 0.000 [obese vs. non-obese] |
|  | Risk of Mortality | Obesity: OR = 1.19 (0.94, 1.51), p = 0.147 [obese vs. non-obese] Morbid obesity (BMI >= 40): OR = 0.98 (0.80-1.20), p=0.858 [morbid obese vs. non-morbid obese] |
| Mahamat-Saleh et al. (2021)[47] | Risk of Mortality | SSR = 1.45 (1.31,1.61) for patients with BMI >= 30 compared with those with BMI <30  Per 5 unit increase in BMI, risk of mortality SSR = 1.12 (1.07, 1.17) -The absolute risk of COVID-19 death increased by 12% for obesity. [obese vs. non-obese] |
| Raeisi et al. (2021)[48] | Risk of COVID-19 Diagnosis | The prevalence of obesity was 33% (30.0% - 35.0%) among patients with COVID-19. OR = 2.42 (1.58, 3.70) [obese vs. non-obese] |
|  | Risk of Severe COVID-19 Disease | OR = 1.62 (1.48,1.76) with low certainty [obese vs. non-obese] |
|  | Risk of COVID-19 Hospitalization | OR = 1.75 (1.47, 2.09) with very low certainty [obese vs. non-obese] |
|  | Risk of ICU Admission | OR = 1.75 (1.38, 2.22) with low certainty [obese vs. non-obese] |
|  | Risk of Mechanical Ventilation | OR = 2.24 (1.70,2.94) with low certainty [obese vs. non-obese] |
|  | Risk of Mortality | OR = 1.23 (1.06, 1.41) with low certainty [obese vs. non-obese] |
| Noor and Islam (2020)[49] | Risk of COVID-19 Hospitalization | RR = 2.18 (1.10,4.34), p<0.05  [obese vs. non-obese] |
| Zhang et al. (2021)[50] | Risk of Severe COVID-19 Disease | OR = 3.13 (1.41,6.92), p=0.005 [obese vs. non-obese] |
|  | Risk of COVID-19 Hospitalization | Prevalence of obesity = 27.6% (22.0, 33.2) |
|  | Risk of ICU Admission | OR = 1.25 (0.99,1.58), p = 0.062 [obese vs. non-obese] |
|  | Risk of Mortality | OR =1.50 (1.25,1.81), p<0.001 [obese vs. non-obese] |
| Rajiva et al. (2021)[51] | Risk of COVID-19 Diagnosis | OR = 2.95 (2.30,3.78) (overweight vs normal BMI) |
| Wood et al. (2021)[52] | Risk of ICU Admission | OR = 1.21 (1,1.46) BMI >25 (overweight vs. normal weight) OR = 2.05 (1.16-3.64) BMI >30 (obese vs. overweight&normal) |
|  | Risk of Mortality | Overweight and obesity vs normal weight: RR = 0.83 (0.77-0.91) |
|  | Other | ICU admission, IMV, or death: Obese vs. non-obese: RR = 1.40 (0.91, 2.17) |
| Li et al. (2021)[53] | Risk of Severe COVID-19 Disease | Obese (BMI >=30) vs. non obese (OR = 1.89 (1.44,2.46) |
| Wilk-Sledziewska et al. (2022)[54] | Risk of ICU Admission | Obese patients <60 years old with BMI >=35 are 3.6 times more likely to be admitted to ICU compared to patients with normal BMI in the same age. |
| Pranata et al. (2021)[55] | Risk of Severe COVID-19 Disease | Obese vs. non-obese: OR = 1.90 (1.45,2.48), p<0.001 A higher BMI in the patients was associated with aOR = 3.08 (1.78,5.33), p<0.001 |
|  | Risk of Mortality | Obese vs. non-obese: OR = 1.55 (1.16,2.06), p=0.003 A higher BMI in the patients was associated with aOR = 2.85 (1.17,6.92), p=0.002 |
|  | Other | Poor outcome: OR = 1.73 (1.40,2.14), p <0.001 A higher BMI in the patients was associated with aOR = 3.02 (1.82,5.00), p<0.001 -A dose-response meta-analysis showed an increased risk of composite poor outcome by aOR of 1.052 (1.028,1.077), p<0.001 for every 5 unit increase in BMI |
| Amato et al. (2021)[56] | Risk of Mortality | Obese vs non-obese: OR = 2.9 (1.1-7.6) |
| Deng et al. (2021)[57] | Risk of COVID-19 Diagnosis | Prevalence of obesity in patients with COVID-19 = 30% (21%,39%) [obese vs. non-obese] |
|  | Risk of Severe COVID-19 Disease | Obese vs non-obese: OR = 1.79 (1.52,2.11), p <0.0001 |
|  | Risk of ICU Admission | OR = 1.20 (1.11,1.30), p<0.0001 [obese vs. non-obese] |
|  | Risk of Mechanical Ventilation | OR = 1.16 (1.10,1.23), p <0.0001 [obese vs. non-obese] |
|  | Risk of Mortality | OR = 1.05 (0.65,1.71), p = 0.84 [obese vs. non-obese] |
| Yanai (2020)[58] | Risk of COVID-19 Diagnosis | incidence of COVID-19 in obese vs non-obese patients: OR = 2.20 (2.10,2.32) |
| Mesas et al. (2020)[59] | Risk of Mortality | Obese vs non-obese: OR = 1.09 (0.84,1.41), p=0.53 |
| Tsankov et al. (2020)[60] | Risk of COVID-19 Diagnosis | Children with obesity had a relative risk ratio of 2.87 (1.16,7.07) |
| Helvaci et al. (2021)[61] | Risk of COVID-19 Hospitalization | Pooled obesity-prevalence rate was 0.32 (0.24,0.41) OR = 1.3 (1.00,1.69), p = 0.05 |
|  | Risk of ICU Admission | Pooled obesity-prevalence rate was 0.41 (0.36,0.45) OR = 1.51 (1.16, 1.97), p = 0.002 |
|  | Risk of Mechanical Ventilation | Pooled obesity-prevalence rate was 0.43 (0.36,0.51) OR = 1.77 (1.34,2.35), p<0.001 |
|  | Risk of Mortality | Pooled obesity-prevalence rate was 0.33 (0.26,0.41) OR = 1.28 (0.76,2.16), p = 0.35 |
| Ho et al. (2020)[62] | Risk of COVID-19 Diagnosis | OR = 1.50 (1.25,1.81), p<0.001 [obese vs. non-obese] |
|  | Risk of Severe COVID-19 Disease | OR = 3.13 (1.41-6.92), p = 0.005 [obese vs. non-obese] |
|  | Risk of COVID-19 Hospitalization | Pooled prevalence of obesity: 27.6% (22.0,33.2) |
|  | Risk of ICU Admission | OR = 1.25 (0.99,1.58), p = 0.062 [obese vs. non-obese] |
|  | Risk of Mortality | OR = 1.36 (1.09,1.69), p = 0.006 [obese vs. non-obese] |
| Michalakis et al. (2021)[63] | Risk of Mechanical Ventilation | OR = (1.20,7.36) overweight or obesity vs normal |
|  | Risk of Mortality | Being overweight or having obesity with COVID-19: OR = (1.22,3.68) |
| Choi, Choi, and Yun (2022)[64] | Risk of Severe COVID-19 Disease | Risk factors for severe COVID-19 in children: (RR = 1.43 (1.24, 1.64)) [obese vs. non-obese] |

**Table C in S1 Tables. Summary of parameter projection sources.**

| **Projection Type** | **Future Parameter Values** | **Source** |
| --- | --- | --- |
| Projection from secondary sources | Population | United Nations Population Division (UNPD) (projections to 2030)[9] |
| ·· | Life expectancy | United Nations Population Division (projections to 2030)[9] |
| ·· | Background death rates | United Nations Population Division (projections to 2030) [9] |
| ·· | Annual GDP for Australia, Brazil, India, Mexico, Saudi Arabia, South Africa, Spain | IMF Economic Outlook – Long-term baseline projections (projections to 2030)[65] |
| ·· | GDP Deflator for Australia, Brazil, India, Mexico, Saudi Arabia, South Africa, Spain | OECD Economic Outlook (Long-term baseline projections to 2030) [65] |
| .. | Overweight and Obesity (OAO) prevalence for ages under and above 20 years and by sex. | NCD Risk Factor Collaboration (NCD-RiSC)[8] |
| ·· | COVID-19 hospitalization, ICU admissions and deaths | IHME Database [66] and OurWorldInData.org [7] |
| Financial parameters adjusted for inflation | Cost per inpatient/hospitalized COVID-19 care and ICU COVID-19 care |  |

**Table D in S1 Tables. Overweight and obesity (OAO) prevalence-historical and projected estimates.**

| **Country** | **Year** | **OAO Prevalence (males over 20 years)*** | **OAO Prevalence (females over 20 years)*** | **OAO Prevalence (males under 20 years)*** | **OAO Prevalence (females under 20 years)*** | **Total OAO Prevalence¶** |
| --- | --- | --- | --- | --- | --- | --- |
| Australia | 1975 | 48.0% | 38.1% | 19.5% | 20.3% | 34.6% |
| Australia | 2016 | 72.9% | 59.8% | 35.6% | 32.6% | 58.2% |
| Australia | 2020 | 76.4% | 63.1% | 39.8% | 36.3% | 61.7% |
| Australia | 2030 | 82.2% | 69.3% | 46.0% | 40.8% | 67.7% |
| Brazil | 1975 | 25.5% | 31.2% | 5.9% | 8.5% | 17.6% |
| Brazil | 2016 | 59.4% | 57.1% | 30.0% | 26.6% | 49.1% |
| Brazil | 2020 | 64.6% | 61.8% | 36.2% | 31.3% | 54.8% |
| Brazil | 2030 | 73.8% | 69.1% | 48.9% | 39.7% | 64.7% |
| India | 1975 | 4.6% | 6.6% | 0.3% | 0.4% | 2.9% |
| India | 2016 | 18.5% | 22.4% | 7.5% | 6.1% | 15.3% |
| India | 2020 | 21.0% | 25.4% | 10.1% | 7.8% | 18.1% |
| India | 2030 | 28.5% | 33.3% | 20.9% | 14.6% | 26.8% |
| Mexico | 1975 | 33.6% | 43.2% | 10.1% | 12.3% | 23.0% |
| Mexico | 2016 | 65.4% | 67.6% | 35.8% | 35.2% | 55.3% |
| Mexico | 2020 | 70.2% | 71.2% | 42.4% | 40.6% | 60.6% |
| Mexico | 2030 | 77.8% | 76.8% | 52.5% | 49.5% | 69.3% |
| Saudi Arabia | 1975 | 34.6% | 44.6% | 5.7% | 8.5% | 21.9% |
| Saudi Arabia | 2016 | 70.1% | 73.7% | 38.1% | 32.1% | 59.7% |
| Saudi Arabia | 2020 | 75.5% | 78.2% | 46.5% | 39.4% | 66.1% |
| Saudi Arabia | 2030 | 82.9% | 83.4% | 60.1% | 49.5% | 75.0% |
| South Africa | 1975 | 16.2% | 40.3% | 1.4% | 3.8% | 15.1% |
| South Africa | 2016 | 42.0% | 67.2% | 20.2% | 29.4% | 43.5% |
| South Africa | 2020 | 46.3% | 70.6% | 26.8% | 35.5% | 48.5% |
| South Africa | 2030 | 57.1% | 76.7% | 55.9% | 59.3% | 63.9% |
| Spain | 1975 | 45.5% | 39.0% | 17.2% | 15.4% | 32.9% |
| Spain | 2016 | 70.9% | 55.8% | 36.7% | 30.7% | 57.4% |
| Spain | 2020 | 74.4% | 58.7% | 42.0% | 33.5% | 60.9% |
| Spain | 2030 | 80.1% | 62.9% | 48.4% | 38.6% | 66.6% |
| Thailand | 1975 | 6.8% | 11.0% | 1.0% | 1.3% | 4.8% |
| Thailand | 2016 | 30.3% | 36.8% | 25.1% | 19.3% | 30.9% |
| Thailand | 2020 | 32.9% | 41.2% | 33.8% | 26.6% | 35.7% |
| Thailand | 2030 | 44.9% | 53.4% | 55.2% | 45.5% | 49.6% |

**Prevalence estimates for 1975-2016 are historical data drawn from NCD RisC; Projected prevalence estimates are from 2017 to 2025.*

*¶Authors’ calculations from prevalence estimates for male and female under and above 20 years of age*

**Works Cited**

1. Institute for Health Metrics and Evaluation (IHME). Global Inpatient and Outpatient Health Care Utilization, Unit Costs, and Costs and Services Needed to Achieve Universal Health Coverage 1990-2016. Institute for Health Metrics and Evaluation (IHME); 2018. doi:10.6069/RW6Y-K168

2. Khan A, AlRuthia Y, Balkhi B, Alghadeer S, Temsah M-H, Althunayyan S, et al. Survival and Estimation of Direct Medical Costs of Hospitalized COVID-19 Patients in the Kingdom of Saudi Arabia. Int J Environ Res Public Health. 2020;17: 7458. doi:10.3390/ijerph17207458

3. Miethke-Morais A, Cassenote AJ, Piva H, Tokunaga E, Cobello V, Alves F, et al. Unraveling COVID-19-related hospital costs: The impact of clinical and demographic conditions. 2020. doi:10.1101/2020.12.24.20248633

4. Kompas T, Grafton RQ, Che TN, Chu L, Camac J. Health and economic costs of early and delayed suppression and the unmitigated spread of COVID-19: The case of Australia. PLOS ONE. 2021;16: e0252400. doi:10.1371/journal.pone.0252400

5. Selvaraj S, Kumar P, Bharali I, Hasan H, Mao W, Ogbuoji O, et al. Costs and Affordability of COVID-19 Testing and Treatment in India. 2021. doi:10.21203/rs.3.rs-703175/v1

6. Cleary SM, Wilkinson T, Tamandjou Tchuem CR, Docrat S, Solanki GC. Cost‐effectiveness of intensive care for hospitalized COVID-19 patients: experience from South Africa. BMC Health Serv Res. 2021;21: 82. doi:10.1186/s12913-021-06081-4

7. Ritchie H, Mathieu E, Rodés-Guirao L, Appel C, Giattino C, Ortiz-Ospina E, et al. Coronavirus Pandemic (COVID-19). Our World Data. 2020 [cited 30 Aug 2021]. Available: https://ourworldindata.org/coronavirus

8. NCD-RisC. Data Downloads: Download files containing country risk factor data. Collaboration NRF, editor. NCD-RisC: NCD Risk Factor Collaboration; 2017. Available: http://ncdrisc.org/data-downloads.html

9. World Population Prospects - Population Division - United Nations. [cited 9 Oct 2020]. Available: https://population.un.org/wpp/

10. Yang J, Hu J, Zhu C. Obesity aggravates COVID-19: A systematic review and meta-analysis. J Med Virol. 93: 257–261. doi:10.1002/jmv.26237

11. Huang Y, Lu Y, YM H, Wang M, Ling W, Sui Y, et al. Obesity in patients with COVID-19: a systematic review and meta-analysis. Metabolism. 113: 154378–154378. doi:10.1016/j.metabol.2020.154378

12. Tamara A, DL T. Obesity as a predictor for a poor prognosis of COVID-19: A systematic review. Diabetes Metab Syndr. 14: 655–659. doi:10.1016/j.dsx.2020.05.020

13. Földi M, Farkas N, Kiss S, Zádori N, Váncsa S, Szakó L, et al. Obesity is a risk factor for developing critical condition in COVID-19 patients: A systematic review and meta-analysis. Obes Rev. 21: e13095–e13095. doi:10.1111/obr.13095

14. Malik V, Ravindra K, SV A, SK B, Singh M. Higher body mass index is an important risk factor in COVID-19 patients: a systematic review and meta-analysis. Env Sci Pollut Res Int. 27: 42115–42123. doi:10.1007/s11356-020-10132-4

15. Chang T, CC C, LY C. Effect of obesity and body mass index on coronavirus disease 2019 severity: A systematic review and meta-analysis. Obes Rev. 21: e13089–e13089. doi:10.1111/obr.13089

16. Sales-Peres S, LJ de A-S, RCS B, MC S-P, ACDS P, JF SJ. Coronavirus (SARS-CoV-2) and the risk of obesity for critically illness and ICU admitted: Meta-analysis of the epidemiological evidence. Obes Res Clin Pr. 14: 389–397. doi:10.1016/j.orcp.2020.07.007

17. Soeroto A, NN S, Purwiga A, Santoso P, ID K, Suryadinata H, et al. Effect of increased BMI and obesity on the outcome of COVID-19 adult patients: A systematic review and meta-analysis. Diabetes Metab Syndr. 14: 1897–1904. doi:10.1016/j.dsx.2020.09.029

18. Seidu S, Gillies C, Zaccardi F, Kunutsor SK, Hartmann-Boyce J, Yates T, et al. The impact of obesity on severe disease and mortality in people with SARS-CoV-2: A systematic review and meta-analysis. Endocrinol Diabetes Metab. 2021;4: e00176. doi:10.1002/edm2.176

19. Malik P, Patel U, Patel K, Martin M, Shah C, Mehta D, et al. Obesity a predictor of outcomes of COVID-19 hospitalized patients-A systematic review and meta-analysis. J Med Virol. 2021;93: 1188–1193. doi:10.1002/jmv.26555

20. Chu Y, Yang J, Shi J, Zhang P, Wang X. Obesity is associated with increased severity of disease in COVID-19 pneumonia: a systematic review and meta-analysis. Eur J Med Res. 2020;25: 64. doi:10.1186/s40001-020-00464-9

21. Yang J, Tian C, Chen Y, Zhu C, Chi H, Li J. Obesity aggravates COVID-19: An updated systematic review and meta-analysis. J Med Virol. 2021;93: 2662–2674. doi:10.1002/jmv.26677

22. Földi M, Farkas N, Kiss S, Dembrovszky F, Szakács Z, Balaskó M, et al. Visceral Adiposity Elevates the Risk of Critical Condition in COVID-19: A Systematic Review and Meta-Analysis. Obes Silver Spring. 29: 521–528. doi:10.1002/oby.23096

23. Zhao X, Gang X, He G, Li Z, Lv Y, Han Q, et al. Obesity Increases the Severity and Mortality of Influenza and COVID-19: A Systematic Review and Meta-Analysis. Front Endocrinol Lausanne. 11: 595109–595109. doi:10.3389/fendo.2020.595109

24. Hoong C, Hussain I, VM A, EE P, JHX L, Koh H. Obesity is Associated with Poor Covid-19 Outcomes: A Systematic Review and Meta-Analysis. Horm Metab Res. 53: 85–93. doi:10.1055/a-1326-2125

25. Shi Q, Wang Z, Liu J, Wang X, Zhou Q, Li Q, et al. Risk factors for poor prognosis in children and adolescents with COVID-19: A systematic review and meta-analysis. EClinicalMedicine. 41: 101155–101155. doi:10.1016/j.eclinm.2021.101155

26. Cai Z, Yang Y, Zhang J. Obesity is associated with severe disease and mortality in patients with coronavirus disease 2019 (COVID-19): a meta-analysis. BMC Public Health. 21: 1505–1505. doi:10.1186/s12889-021-11546-6

27. Zhou Y, Yang Q, Chi J, Dong B, Lv W, Shen L, et al. Comorbidities and the risk of severe or fatal outcomes associated with coronavirus disease 2019: A systematic review and meta-analysis. Int J Infect Dis. 99: 47–56. doi:10.1016/j.ijid.2020.07.029

28. Groah G, CT P, AK R, JJ S. Outcomes of patients with COVID-19 after inpatient rehabilitation. PM R. 14: 202–209. doi:10.1002/pmrj.12645

29. Harrison S, BJR B, JM R-C, Zhang J, GYH L. Cardiovascular risk factors, cardiovascular disease, and COVID-19: an umbrella review of systematic reviews. Eur Heart J Qual Care Clin Outcomes. 7: 330–339. doi:10.1093/ehjqcco/qcab029

30. Popkin B, Du S, WD G, MA B, Algaith T, CH H, et al. Individuals with obesity and COVID-19: A global perspective on the epidemiology and biological relationships. Obes Rev. 21: e13128–e13128. doi:10.1111/obr.13128

31. Dessie Z, Zewotir T. Mortality-related risk factors of COVID-19: a systematic review and meta-analysis of 42 studies and 423,117 patients. BMC Infect Dis. 21: 855–855. doi:10.1186/s12879-021-06536-3

32. Das P, Samad N, AA S, RG A, JK T, BO A. Obesity as a predictor for adverse outcomes among COVID-19 patients: A meta-analysis. medRxiv. doi:10.1101/2020.11.27.20239616

33. Aghili S, Ebrahimpur M, Arjmand B, Shadman Z, M PS, Qorbani M, et al. Obesity in COVID-19 era, implications for mechanisms, comorbidities, and prognosis: a review and meta-analysis. Int J Obes. 45: 998–1016. doi:10.1038/s41366-021-00776-8

34. de Frel D, DE A, Pijl H, JC S, PJM L, WA D, et al. The Impact of Obesity and Lifestyle on the Immune System and Susceptibility to Infections Such as COVID-19. Front Nutr. 7: 597600–597600. doi:10.3389/fnut.2020.597600

35. Hu J, Wang Y. The Clinical Characteristics and Risk Factors of Severe COVID-19. Gerontology. 67: 255–266. doi:10.1159/000513400

36. Taylor O augments the disease burden in C-19: U data from an umbrella review, EJ M, Elhadi M, KDM M, YC Y, Davids R, et al. Factors associated with mortality in patients with COVID-19 admitted to intensive care: a systematic review and meta-analysis. Anaesthesia. 76: 1224–1232. doi:10.1111/anae.15532

37. Rico-Martín S, JF C-G, Basilio-Fernández B, MZ C-C, JF SM-T. Metabolic Syndrome and Its Components in Patients with COVID-19: Severe Acute Respiratory Syndrome (SARS) and Mortality. A Systematic Review and Meta-Analysis. J Cardiovasc Dev Dis. 8. doi:10.3390/jcdd8120162

38. Kristensen D higher body mass index increase C-19 severity? A systematic review and meta-analysis, SB G, AL A, Richelsen B, JM B. Obesity augments the disease burden in COVID-19: Updated data from an umbrella review. Clin Obes. : e12508–e12508. doi:10.1111/cob.12508

39. Booth A, AB R, Ponzo S, Yassaee A, Aral M, Plans D, et al. Population risk factors for severe disease and mortality in COVID-19: A global systematic review and meta-analysis. PLoS One. 16: e0247461–e0247461. doi:10.1371/journal.pone.0247461

40. Chowdhury C, MR A, MF R, Rahman T, Reza S. Does higher body mass index increase COVID-19 severity? A systematic review and meta-analysis. Obes Med. 23: 100340–100340. doi:10.1016/j.obmed.2021.100340

41. Sahu S, Mathew R, Aggarwal P, Nayer J, Bhoi S, Satapathy S, et al. Clinical Determinants of Severe COVID-19 Disease - A Systematic Review and Meta-Analysis. J Glob Infect Dis. 13: 13–19. doi:10.4103/jgid.jgid_136_20

42. Poly P, MM I, HC Y, MC L, WS J, MH H, et al. Obesity and Mortality Among Patients Diagnosed With COVID-19: A Systematic Review and Meta-Analysis. Front Med Lausanne. 8: 620044–620044. doi:10.3389/fmed.2021.620044

43. Xu L, Mao Y, Chen G. Risk factors for 2019 novel coronavirus disease (COVID-19) patients progressing to critical illness: a systematic review and meta-analysis. Aging. 12: 12410–12421. doi:10.18632/aging.103383

44. Li Y, Ashcroft T, Chung A, Dighero I, Dozier M, Horne M, et al. Risk factors for poor outcomes in hospitalised COVID-19 patients: A systematic review and meta-analysis. J Glob Health. 11: 10001–10001. doi:10.7189/jogh.11.10001

45. Du Y, Lv Y, Zha W, Zhou N, Hong X. Association of body mass index (BMI) with critical COVID-19 and in-hospital mortality: A dose-response meta-analysis. Metabolism. 117: 154373–154373. doi:10.1016/j.metabol.2020.154373

46. Geng J, Yu X, Bao H, Feng Z, Yuan X, Zhang J, et al. Chronic Diseases as a Predictor for Severity and Mortality of COVID-19: A Systematic Review With Cumulative Meta-Analysis. Front Med Lausanne. 8: 588013–588013. doi:10.3389/fmed.2021.588013

47. Mahamat-Saleh Y, Fiolet T, ME R, Mulot M, Guihur A, D EF, et al. Diabetes, hypertension, body mass index, smoking and COVID-19-related mortality: a systematic review and meta-analysis of observational studies. BMJ Open. 11: e052777–e052777. doi:10.1136/bmjopen-2021-052777

48. Raeisi T, Mozaffari H, Sepehri N, Darand M, Razi B, Garousi N, et al. The negative impact of obesity on the occurrence and prognosis of the 2019 novel coronavirus (COVID-19) disease: a systematic review and meta-analysis. Eat Weight Disord. 27: 893–911. doi:10.1007/s40519-021-01269-3

49. Noor N, MM I. Prevalence and Associated Risk Factors of Mortality Among COVID-19 Patients: A Meta-Analysis. J Community Health. 45: 1270–1282. doi:10.1007/s10900-020-00920-x

50. Zhang X, AM L, JR M, JR B. A systematic review and meta-analysis of obesity and COVID-19 outcomes. Sci Rep. 11: 7193–7193. doi:10.1038/s41598-021-86694-1

51. Rajiva R, DS F, Bobdey S, MPS P. Overweight: A risk factor for COVID-19 --- A medical conundrum or a reality? J Fam Med Prim Care. 10: 4096–4101. doi:10.4103/jfmpc.jfmpc_889_21

52. Wood S, SE H, Judd N, MA B, Hughes K, Jones A. The impact of behavioural risk factors on communicable diseases: a systematic review of reviews. BMC Public Health. 21: 2110–2110. doi:10.1186/s12889-021-12148-y

53. Li X, Zhong X, Wang Y, Zeng X, Luo T, Liu Q. Clinical determinants of the severity of COVID-19: A systematic review and meta-analysis. PLoS One. 16: e0250602–e0250602. doi:10.1371/journal.pone.0250602

54. Wilk-Sledziewska K, PJ S, Uscinska N, Bujno E, Rosolowski M, Kakareko K, et al. The Impact of Cardiovascular Risk Factors on the Course of COVID-19. J Clin Med. 11. doi:10.3390/jcm11082250

55. Pranata R, MA L, Yonas E, Vania R, AA L, BB S, et al. Body mass index and outcome in patients with COVID-19: A dose-response meta-analysis. Diabetes Metab. 47: 101178–101178. doi:10.1016/j.diabet.2020.07.005

56. Amato J, PM C, FMSB C, Meyer G, LJ P, LC S, et al. Assessing Predictive Factors of COVID-19 Outcomes: A Retrospective Cohort Study in the Metropolitan Region of São Paulo (Brazil). Med Kaunas. 57. doi:10.3390/medicina57101068

57. Deng L, Zhang J, Wang M, Chen L. Obesity is associated with severe COVID-19 but not death: a dose-response meta-analysis. Epidemiol Infect. 149: e144–e144. doi:10.1017/S0950268820003179

58. Yanai H. Metabolic Syndrome and COVID-19. Cardiol Res. 11: 360–365. doi:10.14740/cr1181

59. Mesas M, Cavero-Redondo I, Álvarez-Bueno C, MA SC, S M de A, Sequí-Dominguez I, et al. Predictors of in-hospital COVID-19 mortality: A comprehensive systematic review and meta-analysis exploring differences by age, sex and health conditions. PLoS One. 15: e0241742–e0241742. doi:10.1371/journal.pone.0241742

60. Tsankov B, JM A, MA I, AA L, LJ S, BA V, et al. Severe COVID-19 Infection and Pediatric Comorbidities: A Systematic Review and Meta-Analysis. Int J Infect Dis. 103: 246–256. doi:10.1016/j.ijid.2020.11.163

61. Helvaci N, ND E, Karabulut E, BO Y. Prevalence of Obesity and Its Impact on Outcome in Patients With COVID-19: A Systematic Review and Meta-Analysis. Front Endocrinol Lausanne. 12: 598249–598249. doi:10.3389/fendo.2021.598249

62. Ho J, DI F, MY C, CH S. Obesity in COVID-19: A Systematic Review and Meta-analysis. Ann Acad Med Singap. 49: 996–1008. doi:10.47102/annals-acadmedsg.2020299

63. Michalakis K, Panagiotou G, Ilias I, Pazaitou-Panayiotou K. Obesity and COVID-19: A jigsaw puzzle with still missing pieces. Clin Obes. 11: e12420–e12420. doi:10.1111/cob.12420

64. Choi J, SH C, KW Y. Risk Factors for Severe COVID-19 in Children: A Systematic Review and Meta-Analysis. J Korean Med Sci. 37: e35–e35. doi:10.3346/jkms.2022.37.e35

65. OECD. Economic Outlook No 103 - July 2018 - Long-term baseline projections. In: OECD.Stat [Internet]. [cited 10 Dec 2020]. Available: https://stats.oecd.org/Index.aspx?DataSetCode=EO103_LTB

66. Global Burden of Disease Study. GBD Results Tool | GHDx. [cited 24 Nov 2021]. Available: http://ghdx.healthdata.org/gbd-results-tool
